# Optimization and validation of multi-echo, multi-contrast SAGE acquisition in fMRI

**DOI:** 10.1162/imag_a_00217

**Published:** 2024-07-02

**Authors:** Elizabeth G. Keeling, Maurizio Bergamino, Sudarshan Ragunathan, C. Chad Quarles, Allen T. Newton, Ashley M. Stokes

**Affiliations:** Barrow Neuroimaging Innovation Center, Barrow Neurological Institute, Phoenix, AZ, United States; School of Life Sciences, Arizona State University, Tempe, AZ, United States; Hyperfine, Inc., Guilford, CT, United States; The University of Texas MD Anderson Cancer Center, Houston, TX, United States; Vanderbilt University Institute of Imaging Science, Nashville, TN, United States

**Keywords:** functional MRI (fMRI), multi-echo fMRI, spin-echo fMRI, BOLD contrast, BOLD contrast-to-noise ratio (CNR)

## Abstract

The purpose of this study was to optimize and validate a multi-contrast, multi-echo fMRI method using a combined spin- and gradient-echo (SAGE) acquisition. It was hypothesized that SAGE-based blood oxygen level-dependent (BOLD) functional MRI (fMRI) will improve sensitivity and spatial specificity while reducing signal dropout. SAGE-fMRI data were acquired with five echoes (2 gradient-echoes, 2 asymmetric spin-echoes, and 1 spin-echo) across 12 protocols with varying acceleration factors, and temporal SNR (tSNR) was assessed. The optimized protocol was then implemented in working memory and vision tasks in 15 healthy subjects. Task-based analysis was performed using individual echoes, quantitative dynamic relaxation times T_2_^*^and T_2_, and echo time-dependent weighted combinations of dynamic signals. These methods were compared to determine the optimal analysis method for SAGE-fMRI. Implementation of a multiband factor of 2 and sensitivity encoding (SENSE) factor of 2.5 yielded adequate spatiotemporal resolution while minimizing artifacts and loss in tSNR. Higher BOLD contrast-to-noise ratio (CNR) and tSNR were observed for SAGE-fMRI relative to single-echo fMRI, especially in regions with large susceptibility effects and for T_2_-dominant analyses. Using a working memory task, the extent of activation was highest with T_2_^*^-weighting, while smaller clusters were observed with quantitative T_2_^*^and T_2_. SAGE-fMRI couples the high BOLD sensitivity from multi-gradient-echo acquisitions with improved spatial localization from spin-echo acquisitions, providing two contrasts for analysis. SAGE-fMRI provides substantial advantages, including improving CNR and tSNR for more accurate analysis.

## 1 Introduction

Functional MRI (fMRI) is widely used to map synchronous fluctuations in brain activity via sensitivity to the blood oxygen level-dependent (BOLD) effect. The most common sequence used for fMRI leverages a gradient-echo (GRE) acquisition with echo planar imaging (EPI) readout, which can provide full brain coverage, adequate signal-to-noise ratio (SNR), good temporal resolution, and high sensitivity to BOLD T_2_^*^relaxation effects. The drawbacks of standard fMRI methods include susceptibility-induced signal dropouts near air-tissue interfaces, sensitivity to large draining vessels, and sub-optimal T_2_^*^sensitivity across the brain. This has led to the development of both multi-gradient-echo (MGE) fMRI (Cohen, Jagra, et al., 2021;Cohen et al., 2018;Evans et al., 2015;Kundu et al., 2013;Poser et al., 2006;Posse et al., 1999;Speck & Hennig, 1998) and spin-echo (SE) fMRI methods (Binney et al., 2010;Embleton et al., 2010;Glielmi et al., 2010;Lee et al., 1999), as well as ultra-high-resolution fMRI methods (Knudsen et al., 2023).

MGE-fMRI acquisitions have become more widely adopted in studies of task-based activation and functional connectivity in recent years. MGE-fMRI methods overcome many of the challenges associated with single GRE-EPI. In particular, they are less sensitive to susceptibility effects due to the inclusion of shorter echo times (TE) (Kundu et al., 2013). Additionally, given that the relaxation time T_2_^*^varies greatly across the brain (Hagberg et al., 2002) and the BOLD contrast is maximized when the TE equals the local tissue T_2_^*^, acquiring multiple TEs increases BOLD sensitivity (Poser et al., 2006). Moreover, further signal-to-noise (SNR) benefits are gained by optimally combining echo signals (Kundu et al., 2013). Similarly to a single GRE acquisition, MGE-fMRI is sensitive to large draining vessels (Boxerman et al., 1995). Because the BOLD-associated metabolic exchange is localized in the microvasculature, GRE acquisitions are limited in their spatial specificity. A multi-contrast acquisition could be a logical extension of multi-echo fMRI, pairing the benefits of MGE-fMRI with spatial specificity.

Specifically, spin-echo (SE) EPI could provide a complementary analysis to MGE-fMRI given its sensitivity to the microvasculature (Boxerman et al., 1995). Due to the reduced sensitivity to macrovascular signal contributions that are downstream of neuronal activation, SE methods may improve spatial localization of fMRI activation. In addition, SE acquisitions allow for refocusing of susceptibility-induced signal dropouts, which may be particularly advantageous for characterizing activation in regions near susceptibility interfaces such as inferior frontal and temporal lobes (Norris et al., 2002). SE methods are limited by lower BOLD sensitivity and thus suffer from diminished contrast-to-noise ratio (CNR) (Parkes et al., 2005), in large part preventing SE-fMRI from becoming the gold-standard. Therefore, a combined MGE and SE acquisition could provide complementary information that is useful for comprehensive functional analyses.

Combined MGE and SE acquisitions have been proposed for quantification of relaxation times (Jin et al., 2013;Küppers et al., 2022;Ni et al., 2017;Schmiedeskamp, Straka, Newbould, et al., 2012;Stokes et al., 2014), as well as for simultaneous assessment of cerebral blood flow and single-echo BOLD fMRI task-based activation (Glielmi et al., 2010). Additionally, T_2_-weighted gradient- and spin-echo (GRASE) acquisitions have been implemented in laminar fMRI (De Martino et al., 2013;Olman et al., 2012;Oshio & Feinberg, 1991). To our knowledge, a combined MGE and SE acquisition has not been evaluated for use in relaxation time quantification and multi-echo analysis for task-based BOLD fMRI. Presently, we propose the implementation of a combined spin- and gradient-echo (SAGE) sequence, with multiple echoes and contrasts, to unite the advantages of MGE- and SE-fMRI. The SAGE sequence has been previously developed and applied in perfusion MRI, most prominently in neuro-oncological and neuro-vascular pathologies (Schmiedeskamp, Straka, Newbould, et al., 2012;Schmiedeskamp et al., 2013;Skinner et al., 2014;Stokes et al., 2014). For perfusion MRI, SAGE provides advantages over standard single GRE sequences, including reduced sensitivity to T_1_effects (due to contrast leakage in perfusion MRI (Stokes, Semmineh, et al., 2016)), optimal TEs for different tissues (Bell et al., 2017), and both macro- and microvascular sensitivity (Schmiedeskamp et al., 2013). Using the SAGE sequence, both relaxation rates R_2_^*^(=1/T_2_^*^) and R_2_(=1/T_2_) can be quantified (Schmiedeskamp, Straka, & Bammer, 2012;Stokes & Quarles, 2016;Stokes et al., 2014). In the context of fMRI, quantitative R_2_^*^(and R_2_) may be more directly related to neuronal activation via the susceptibility differences between oxygenated and deoxygenated blood than a single T_2_^*^-weighted GRE given the ability to parse BOLD and non-BOLD components of a signal with multiple echoes (Buxton, 2013;Poser et al., 2006;Speck & Hennig, 1998). Specifically, quantitative R_2_^*^(and R_2_) may permit separation of the complex factors (both BOLD-based and non-BOLD-based, such as cardiac and respiratory noise and head motion) that contribute to fMRI signals (Kundu et al., 2012). Moreover, multi-echo and multi-contrast combinations using weighted summation strategies, which involve combination of signals across multiple TEs for optimized analysis, may provide further benefit for fMRI analysis (Poser et al., 2006;Posse et al., 1999). While multi-echo combinations offer many advantages, acquisitions require acceleration in order to meet the spatiotemporal resolution demands of specific research questions. Acceleration must be balanced with changes in signal-to-noise ratio, as well as ideal TEs based on tissue sensitivities.

The purpose of this study was two-fold: 1) to optimize the acquisition protocol by quantitatively assessing acceleration factors for SAGE in fMRI, as prior implementations have focused on perfusion MRI, and 2) validate SAGE-fMRI in task-based fMRI paradigms, comparing results to single-echo methods. For this purpose, we acquired dynamic SAGE-fMRI data with five echoes (2 GRE, 2 asymmetric spin-echoes (ASE), and 1 SE), first in 6 healthy subjects for optimization, then in 15 healthy subjects for validation using working memory and visual stimulus tasks. Single-echo analyses using the second and fifth echoes (i.e., analogous to standard GRE- and SE-fMRI, respectively) were compared to multi-echo SAGE analyses, which included quantitative dynamic T_2_^*^and T_2_maps and relative relaxation-weighted dynamic signals. We hypothesized that SAGE-based BOLD fMRI (Schmiedeskamp et al., 2014) would improve sensitivity and CNR, while reducing signal dropout through the inclusion of multiple echoes and contrasts.

## 2 Methods

### 2.1 SAGE-fMRI theory

The standard SAGE sequence includes five echoes, including two GRE, two ASE, and one SE, as shown in Supplementary MaterialsFigure S1A. The SAGE signals at each TE depend on relaxation rates R_2_^*^and/or R_2_, as follows (Stokes et al., 2014):

$$S\left(TE\right)=\{\begin{array}{c} S_{0}^{I}\;\;\cdot \;\;exp\left[-TE\;\;\cdot \;\;R_{2}^{*}\right]\;\;\;\;\;\;\;\;\;\;\;\;\;\;\;\;\;\;\;\;\;\;\;\;\;\;\;\;0<TE<\frac{TE_{SE}}{2}\\ S_{0}^{II}\;\;\cdot \;\;exp\left[-TE_{SE}\;\;\cdot \;\;\left(R_{2}^{*}-R_{2}\right)\right]\;\;\cdot \;\;exp\left[-TE\;\;\cdot \;\;\left(2R_{2}-R_{2}^{*}\right)\right]\;\;\;\;\;\;\;\;\frac{TE_{SE}}{2}<TE<TE_{SE} \end{array}$$ [1]

where$S_{0}^{I}$and$S_{0}^{II}$represent the signal intensities following the excitation and refocusing pulses, respectively, and*TE_SE_*is the TE of the SE. Signals before$\frac{TE_{SE}}{2}$are T_2_*-weighted, whereas signals after$\frac{TE_{SE}}{2}$are of mixed contrast (i.e., ASE TE_3,4_) or T_2_-weighted. The$S_{0}$terms vary due to slice profile mismatch and pulse imperfections (Schmiedeskamp, Straka, & Bammer, 2012). The GREs depend on R_2_^*^, the ASEs depend on both R_2_^*^and R_2_, and the SE depends only on R_2_.

For fMRI, SAGE data can be processed using either the individual echo signals (five echoes with signals*S_1_*-*S_5_*) or multi-echo combinations. For multi-echo sequences, dynamic relaxation rates R_2_^*^and R_2_can be quantified and utilized in subsequent fMRI analyses. Additionally, previous MGE studies have described a weighted multi-echo combination method; this approach has shown the benefit of weighting the signal by the measured relaxation rate, which is effectively the relative contribution to BOLD contrast (Poser et al., 2006;Posse et al., 1999). More specifically, the relaxation-weighting factors can be determined from the derivative of the signal by the specific relaxation rate. Relaxation-weighting produces two separate signals for analysis, one each for T_2_^*^-weighting and T_2_-weighting, by combining the five SAGE-fMRI signals with specific weighting factors. Thus, the relative relaxation-weighting factors for T_2_^*^and T_2_(*w*T_2_^*^and*w*T_2_, respectively) are given by the derivative ofEq. 1:

$$wT_{2}^{*}\left(TE\right)=\{\begin{array}{c} TE\;\;\cdot \;\;exp\left[-TE\;\;\cdot \;\;R_{2}^{*}\right]\;\;\;\;\;\;\;\;\;\;\;\;\;\;\;\;\;\;\;\;\;\;\;\;\;0<TE<\frac{TE_{SE}}{2}\\ \begin{array}{c} \left(TE_{SE}-TE\right)\;\;\cdot \;\;exp\left[-TE_{SE}\left(R_{2}^{*}-R_{2}\right)-TE\left(2R_{2}-R_{2}^{*}\right)\right]\; \end{array}\;\;\;\;\;\;\frac{TE_{SE}}{2}<TE<TE_{SE} \end{array}$$ [2]

$$wT_{2}\left(TE\right)=\{\begin{array}{c} \;\;\;\;\;\;\;\;\;\;\;0\;\;\;\;\;\;\;\;\;\;\;\;\;\;\;\;\;\;\;\;\;\;\;\;\;\;\;\;\;0<TE<\frac{TE_{SE}}{2}\\ \begin{array}{c} (2TE-TE_{SE})\;\;\cdot \;\;exp\left[-TE_{SE}\left(R_{2}^{*}-R_{2}\right)-TE\left(2R_{2}-R_{2}^{*}\right)\right]\; \end{array}\;\;\;\;\;\;\frac{TE_{SE}}{2}<TE<TE_{SE} \end{array}\;\;\;\;\;\;$$ [3]

where contributions to each weighting factor are determined by each TE’s contrast (i.e., T_2_^*^, T_2_, or mixed). For simplicity, the signal intensities ($S_{0}^{I}$and$S_{0}^{II}$) and normalization factors$\left(\Sigma_{n}w_{x}(TE_{n})\right)$, wherex= T_2_^*^or T_2_, have been omitted fromEqs. 2to3. The voxel-wise weighting factors for each SAGE signal are shown in Supplementary MaterialsFigure S1B.

#### 2.2.1 Data acquisition: SAGE-fMRI optimization

Dynamic SAGE EPI data were acquired in healthy subjects (n = 6, 33.0 ± 8.7 years old, 1 male) at 3T (Ingenia, Philips) with a 32-channel head coil. The protocol was approved by the local Institutional Review Board (IRB), and all participants provided written informed consent. T_1_-weighted (T_1_-w) anatomical images were acquired using a three-dimensional magnetization-prepared rapid acquisition gradient echo (MPRAGE) sequence with the following acquisition parameters: TR/TE: 7.0/3.2 ms; field of view: 256 × 256 mm^2^; voxel size: 1.0 × 1.0 × 1.0 mm^3^; sagittal slices: 176; and flip angle: 9°. Dynamic SAGE data were acquired with the following acquisition parameters: TR: 3.0 s, field of view: 240 × 240 mm^2^, voxel size: 3.0 × 3.0 × 3.0 mm^3^, and volumes: 40. A reverse phase encoding acquisition was collected for distortion correction. Acceleration factors sensitivity encoding (SENSE) and multiband (MB) influenced the TEs and number of axial slices acquired, while the partial Fourier (PF) factor was held relatively constant (Skinner et al., 2014) (Table 1). The SENSE factor was varied at 2, 2.5, and 3, and the MB factor was varied at 1 (no MB), 2, 3, and 4. For protocol comparisons, TR was held constant at 3.0 s.

**Table 1.** Acquisition parameters across MB and SENSE factors.

MB factor	SENSE factor	TE _1-5_ (ms)	Slices	Z coverage (mm)	PF
1	2	8.4/27/52/70/89	24	72	0.74
	2.5	7.3/22/44/59/74	24	72	0.74
	3	6.4/19/36/49/61	24	72	0.74
**2**	2	9.3/31/66/88/110	36	108	0.74
	**2.5**	**8.0/27/59/78/97**	**36**	**108**	**0.74**
	3	8.0/27/59/78/97	36	108	0.74
3	2	8.9/31/66/88/110	36	108	0.74
	2.5	7.5/27/58/77/96	36	108	0.72
	3	7.4/26/57/76/95	36	108	0.71
4	2	8.9/33/72/95/119	36	108	0.69
	2.5	8.2/30/67/90/112	36	108	0.71
	3	7.6/29/65/86/107	36	108	0.70

The optimal sequence is in bolded text. While most MB factors ≥2 permit full brain coverage (≥120 mm), the number of slices was limited to 36 for comparisons across acceleration factors. Wherever possible, all factors outside of MB and SENSE were held constant for consistency. PF = Partial Fourier.

#### 2.2.2 Data acquisition: SAGE-fMRI validation

Data were acquired in healthy subjects (n = 15, 24.4 ± 2.6 years old, 5 males) at 3T (Ingenia, Philips) with a 32-channel head coil. An estimate of minimum sample size necessary to achieve statistical significance (α= 0.05, power = 0.80, effect size = 0.80) was performed*a priori*using G*Power (Faul et al., 2007) and implemented presently. The protocol was approved by the local IRB, and all participants provided written informed consent. T_1_-w anatomical images were acquired as described above. T_2_-weighted anatomical images were acquired using a fluid attenuated inversion recovery (FLAIR) sequence with the following acquisition parameters: TR/TI: 11000/2800 ms; TE: 125 ms; field of view: 240 × 240 mm^2^; reconstruction matrix: 224 × 224; voxel size: 1.0 × 1.0 × 3.0 mm^3^; and axial slices: 70. BOLD-fMRI data were acquired with the optimized SAGE acquisition. Specifically, acquisition parameters were as follows: SENSE: 2.5; MB: 2; TE_1-5_: 8.0/27/59/78/97 ms; TR: 3.0 s; field of view: 240 × 240 mm^2^; voxel size: 3.0 × 3.0 × 3.0 mm^3^; axial slices: 40; and volumes: 112 (working memory task) and 72 (visual stimulus task). A reverse phase encoding acquisition was collected for distortion correction.

Two tasks were implemented to validate the use of SAGE-fMRI: working memory and visual stimulus. These tasks were chosen to assess the performance of SAGE-fMRI in a range of brain regions. The*N*-back task was implemented to evoke activation in regions associated with working memory (Barch et al., 2013;Drobyshevsky et al., 2006;Marshall et al., 2004;Ragland et al., 2002). This task has shown robust activation in the dorsolateral and anterior prefrontal, inferior frontal, anterior cingulate, and dorsal parietal cortices (Barch et al., 2013;Drobyshevsky et al., 2006). These were considered regions of interest (ROI) in the fMRI analysis and results. A block design was used with 2-back versus 0-back blocks, where the 0-back task was the baseline condition. For the 2-back task, subjects were instructed to click the response device when the letter on screen matched the one that appeared two letters previously; there were 2 matches per block. For the 0-back task, subjects were instructed to click the response device when a specified letter appeared on the screen; there were 3 matches per block. Stimuli appeared for 2 s. Blocks were 30 s each, and there were 10 blocks (5 per condition) in the run.

In a single subject, SAGE-fMRI and standard single-echo GRE and SE acquisitions were acquired during the*N*-back task. All fMRI parameters matched those described above for SAGE-fMRI, except TE and TR were set to 30 ms and 1.5 s, respectively, for GRE, and the SE TE was set to 80 ms (with 3 s TR). Due to the shorter TR, 225 dynamics were acquired for the GRE, yielding the same overall scan time. A reverse phase encoding acquisition was collected for distortion correction. Data were processed as described below, thus enabling direct comparisons between TE_2_and GRE and between TE_5_and SE. As the GRE acquisition had twice as many dynamic volumes, the data were analyzed using both all volumes and with only the odd volumes, enabling a more direct comparison to SAGE-fMRI.

The visual task entails a flashing (8 Hz) radial checkerboard paradigm, previously shown to robustly activate the visual network (Drobyshevsky et al., 2006;Schneider et al., 1993). Subjects were instructed to keep their eyes open for the duration of the task. The task was organized in a block design, where the baseline condition was a crosshair. Blocks were 21 s each, and there were 10 blocks (5 per condition) in the run.

#### 2.3.1 Data pre-processing: SAGE-fMRI optimization

For the optimization portion of this study, the signals from each SAGE acquisition underwent pre-processing to correct for motion (using TE_2_correction for all 5 TEs,*3dvolreg*, AFNI (Cox, 1996)) and distortion (*topup*,*applytopup*, FSL (Jenkinson et al., 2012)). Root mean square (RMS) was calculated for translational and rotational motion; any acquisitions exceeding RMS = 0.3 were excluded from analysis. FreeSurfer (Fischl, 2012) analysis (*recon-all*) was performed for all subjects on anatomical T_1_- and T_2_-weighted images to extract gray and white matter masks for subsequent analysis.

#### 2.3.2 Data pre-processing: SAGE-fMRI validation

For task-based fMRI, each of the five SAGE echoes underwent standard preprocessing. The first two TRs were removed for the signal to reach steady state (these TRs provided instructions to the subject and were therefore not part of the ON-task or baseline conditions;*3dTcat*, AFNI). Data were motion corrected and distortion corrected as described above, followed by de-spiking (*3dDespike*, AFNI), slice-timing correction (*3dTshift*, AFNI), and brain-extraction (*bet*, FSL).

### 2.4 Echo time combinations

SAGE-fMRI data were processed using both single-echo and multi-echo combinations using an in-house MATLAB script (version R2018b, MathWorks, Natick, MA, United States), with a total of nine combinations (5 single-echo and 4 multi-echo). More specifically, each TE was processed individually (five echoes with signals*S_1_*-*S_5_*), where*S_2_*(TE = 27 ms) is the most similar to a standard (single-echo) BOLD-fMRI acquisition and*S_5_*(TE = 97 ms) is similar to single-echo SE-fMRI. SAGE TE_2_and TE_5_, therefore, were included in subsequent statistical analyses for comparison to multi-echo data. For multi-echo analysis, both quantitative fitting of relaxation rates and relaxation-weighted summations were investigated. First, dynamic relaxation rates R_2_^*^and R_2_were quantified by linear least-squares fitting of the log-transformed signals across all SAGE echoes (Eq. 1) (Sisco et al., 2022). An upper threshold of 500 ms was applied to SAGE T_2_^*^- and T_2_-weighted maps to remove outlier voxels; average T_2_^*^and T_2_values for the optimized SAGE-fMRI protocol are reported in Supplementary MaterialsTable S1. Second, weighting strategies (i.e.,*w*T_2_^*^and*w*T_2_) were implemented only for the validation portion of this study, usingEqs. 2and3. Weighting is higher near air-tissue interfaces at shorter TEs, which may improve quantification in these regions (Supplementary MaterialsFig. S1B). For T_2_^*^-weighting, the non-exponential term (Eq. 2) cancels for S_5_, yielding zero contribution as expected due to the complete refocusing of T_2_^*^effects. For T_2_-weighting, there is no BOLD contribution prior to application of the refocusing pulse.

#### 2.5.1 Statistical analysis: SAGE-fMRI optimization

To compare the various single- and multi-echo analysis methods, temporal SNR (tSNR) was calculated for each TE across optimization protocols. The tSNR was calculated voxel-wise using the mean signal intensity$\bar{S}$over time divided by the standard deviation (SD) of the signal$\left(\sigma_{S}\right)$:

$$tSNR=\;\frac{\bar{S}}{\sigma_{S}}$$. [4]

Analysis of variance (ANOVA) was implemented to compare tSNR across MB and SENSE factors. Post-hoc analysis (Tukey’s HSD test) was performed to compare MB and SENSE protocols that exhibited satisfactory acquisition parameters (i.e., whole brain coverage ≥120 mm and TE_5_< 100 ms).

To assess loss in SNR due to acceleration, relative g-factor maps were calculated by dividing the product of the SNR of the least accelerated images (in this study, MB1-SENSE2 is the reference protocol) and the square root of its acceleration factor (R) by the product of the SNR of the higher acceleration images and the square root of its R (Robson et al., 2008):

$$g_{factor}=\;\frac{SNR_{reference}\cdot \sqrt{R_{reference}}}{SNR_{accelerated}\cdot \sqrt{R_{accelerated}}}$$. [5]

SNR was calculated using the difference method, as previously described (Dietrich et al., 2007;Reeder et al., 2005), where the mean whole brain signal from the sum of two images (even and odd dynamics) was divided by the standard deviation of the difference of the two images (Krüger et al., 2001).

Therefore, tSNR and g-factor were used, along with qualitative image quality assessment, full brain coverage (≥120 mm), and sufficient TEs (i.e., TE_1_< 10 ms and TE_5_< 100 ms,), to determine the optimal protocol for whole-brain task-based fMRI.

#### 2.5.2 Statistical analysis and data post-processing: SAGE-fMRI validation

For task-based fMRI, six maps were assessed to directly compare single GRE and single SE fMRI to multi-echo methods: TE_2_, TE_5_, T_2_^*^, T_2_,*w*T_2_^*^, and*w*T_2_; additionally, task-based activation and tSNR results for SAGE TE_1,3-4_are provided in theSupplementary Materials.

tSNR was calculated as the mean signal divided by the standard deviation of the noise time series (i.e., unexplained signal from the general linear model;$\sigma_{noise}$):

$$tSNR=\;\frac{\bar{S}}{\sigma_{noise}}$$. [6]

A*t*-test with pairwise comparisons was performed between T_2_^*^- and T_2_-dominant tSNR maps; results were corrected by cluster size (*3dFWHM*,*3dClustSim*, AFNI) and for multiple comparisons within each map (False Discovery Rate (FDR) <0.05) and across tests (Bonferroni correction).

In addition to tSNR, CNR was calculated, defined as the difference of the signal during stimulus ON and baseline OFF divided by$\sigma_{noise}$(Geissler et al., 2007):

$$CNR=\;\frac{\Delta S}{\sigma_{noise}}$$. [7]

Effect size, specifically Hedge’s*g*, was calculated for working memory and visual stimulus tasks (R version 3.6.3,https://www.R-project.org/, RStudio version 1.4.1717,http://www.rstudio.com/. large effect at*g*≥ 0.80). Hedge’s*g*is defined as a standardized effect size for measuring the difference between two group means and is calculated as the difference of the signal during stimulus ON and baseline OFF, divided by the SD of the signal$\sigma_{S}$:

$$g=\;\frac{\Delta S}{\sigma_{S}}$$. [8]

For task-based fMRI, each single- and multi-echo combination generated from the SAGE BOLD-fMRI acquisition was co-registered to the subject’s anatomical T_1_-w MPRAGE through an applied affine transformation performed on TE_2_(degrees of freedom = 12,*flirt*; FSL). Anatomical images were brain-extracted prior to co-registration (ROBEX (Iglesias et al., 2011)). Subsequently, all SAGE-derived images from each subject were linearly normalized to the MNI space using the co-registration matrix obtained from normalization between MPRAGE and the MNI standard image (*flirt*; FSL). The SAGE-derived images in MNI space were smoothed with a 5.0 mm full-width half-maximum Gaussian blur (*3dmerge*; AFNI) and scaled to have an average fMRI intensity of 100 (*3dTstat*,*3dcalc*, AFNI). Statistical parametric maps of response to stimuli from SAGE-derived images were obtained (*3dDeconvolve*, AFNI), and group analysis was performed (*3dttest++*, AFNI). ROI-based analyses were conducted by using the Harvard Oxford Cortical and Subcortical Atlases (Desikan et al., 2006) and the MNI Structural Atlas (Collins et al., 1995;Mazziotta et al., 2001), using a 50 percent probability threshold. For the working memory task, the following ROIs were included: frontal pole, inferior frontal gyrus (pars triangularis and opercularis), middle frontal gyrus, parietal lobe, cingulate gyrus (anterior and posterior divisions), and frontal medial cortex (Barch et al., 2013;Drobyshevsky et al., 2006). Task-based activation is expected in the frontal pole, inferior frontal gyrus, middle frontal gyrus, and parietal lobe, while default mode network-associated regions (Raichle, 2015) in the cingulate gyrus and frontal medial cortex are expected to be suppressed, showing deactivation. Additionally, the frontal medial cortex ROI allows for comparison of SAGE methods to single-echo fMRI in a region prone to susceptibility effects. For the visual stimulus task, the following ROIs were included: occipital pole, occipital fusiform gyrus, lateral occipital cortex (superior and inferior divisions), and temporal occipital fusiform cortex. To assess differences in GRE- and SE-based functional activation, task-based activation maps were compared in native fMRI space. The Venous Neuroanatomy probabilistic atlas (VENAT) was co-registered into native space and used as a reference for the location of large draining veins in the brain to observe whether macro- and microvascular-weighted methods show significant activation within these veins (Huck et al., 2019).

## 3 Results

### 3.1 SAGE-fMRI: Optimization

**Fig. 1.**
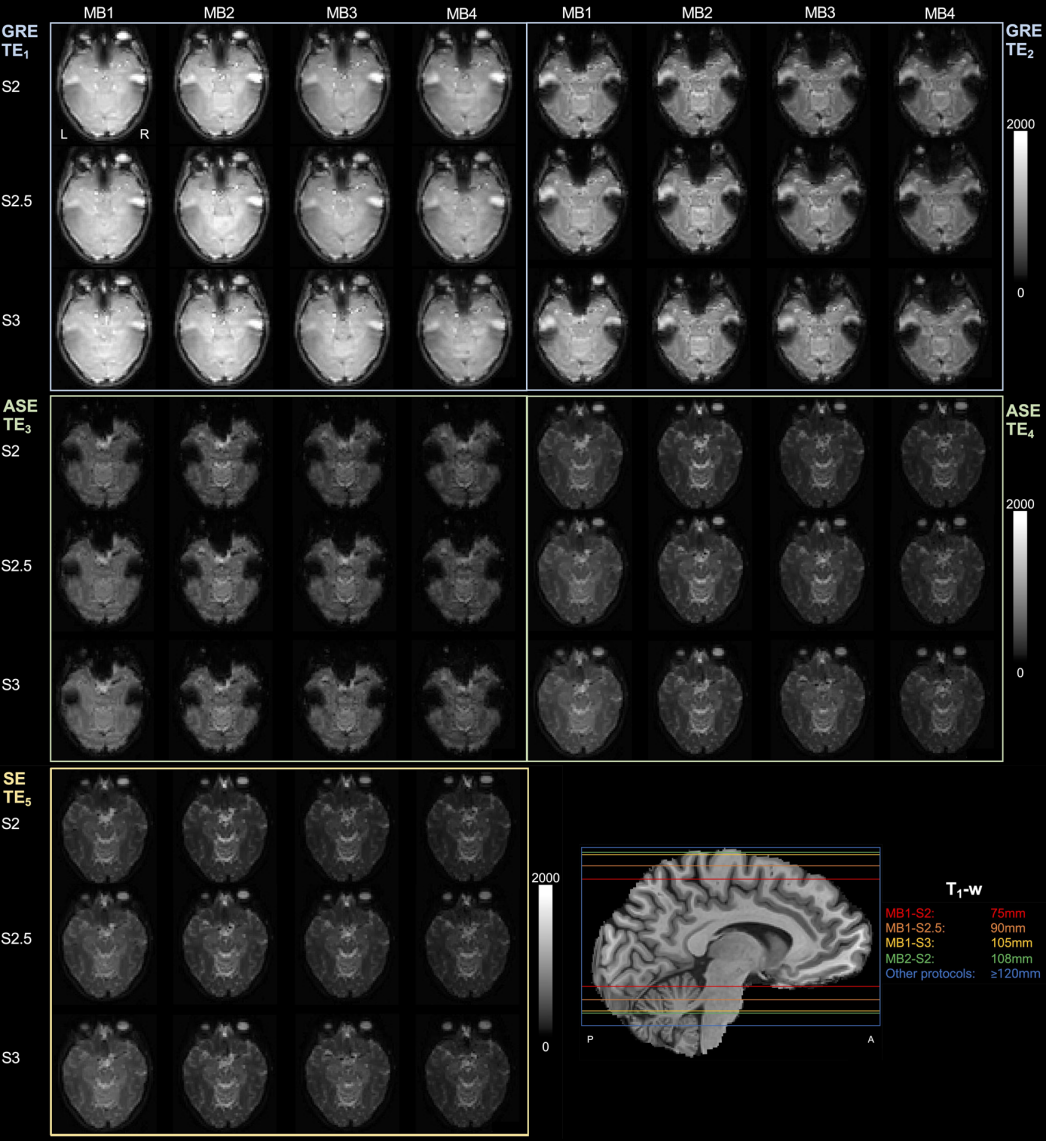
Single-subject acquisition for each TE across acceleration factor combinations. T1-weighted (T1-w) sagittal image is included with each maximum brain coverage achievable per protocol listed in the legend.

Figure 1shows the SAGE signals in a single slice across various combinations of MB and SENSE factors. Without MB (with SENSE = 2 and PF = 0.74), only 75 mm brain coverage was achieved. MB factors 2, 3, and 4 were implemented to enable full brain coverage (i.e., ≥120 mm), with SENSE factors used to ensure adequate TEs (i.e., TE_1_< 10 ms and TE_5_< 100 ms, although TE_5_< 120 ms was permitted to be able to test relevant acceleration factors;Table 1). Qualitative analysis revealed loss of signal and image quality in MB4 across SENSE factors; brain coverage achieved by each protocol is shown in the bottom right sub-panel, where the four least accelerated protocols did not yield full coverage.

**Fig. 2.**
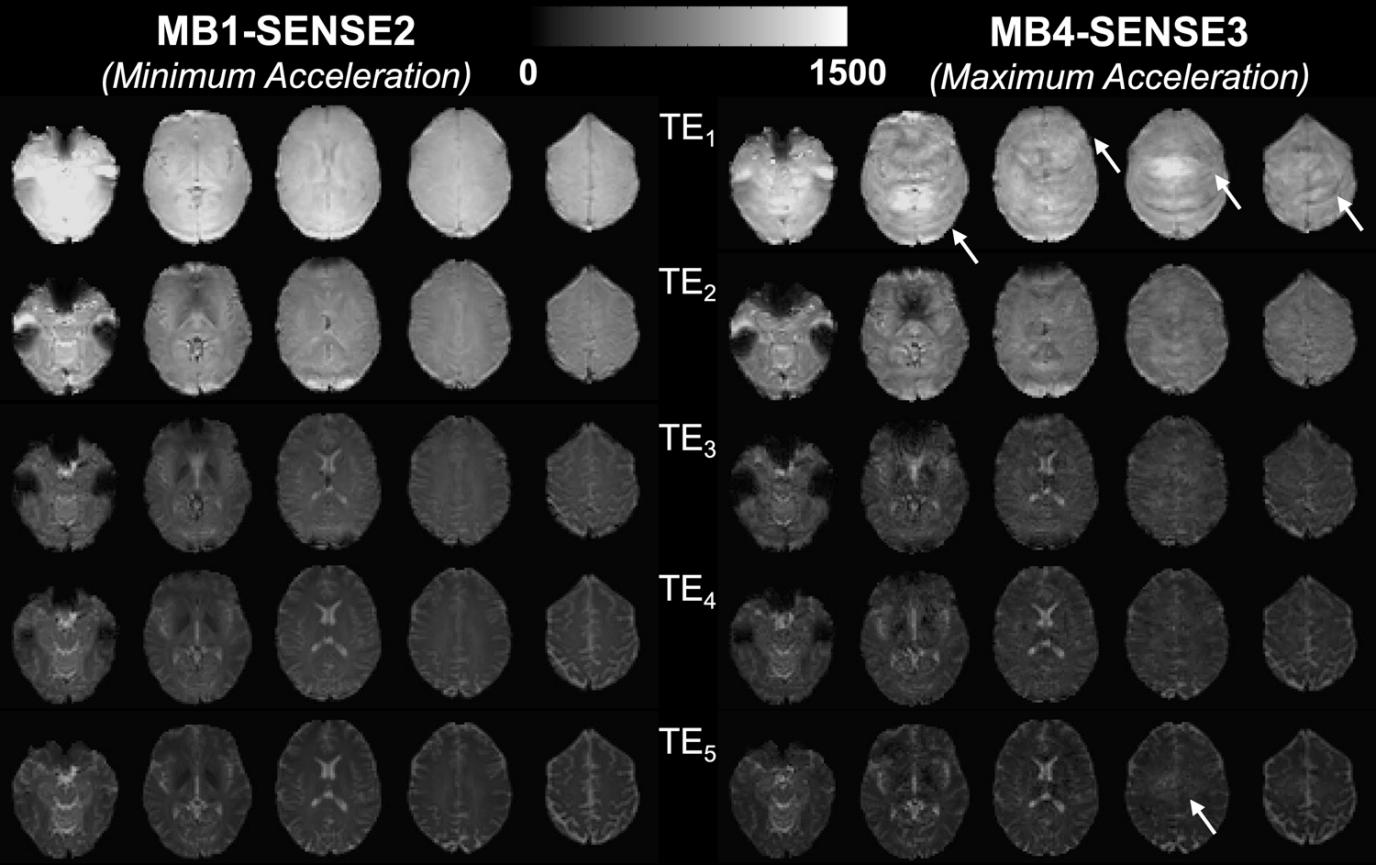
Representative SAGE images for minimum and maximum acceleration protocols per TE. Arrows highlight visible acceleration-related artifacts, apparent in TE_1_as bands and in TE_5_as loss of definition and blurring.

Figure 2shows the loss in image quality from minimum to maximum acceleration protocols in a single subject, where the arrows highlight examples of visible artifacts in the most accelerated acquisition.

**Fig. 3.**
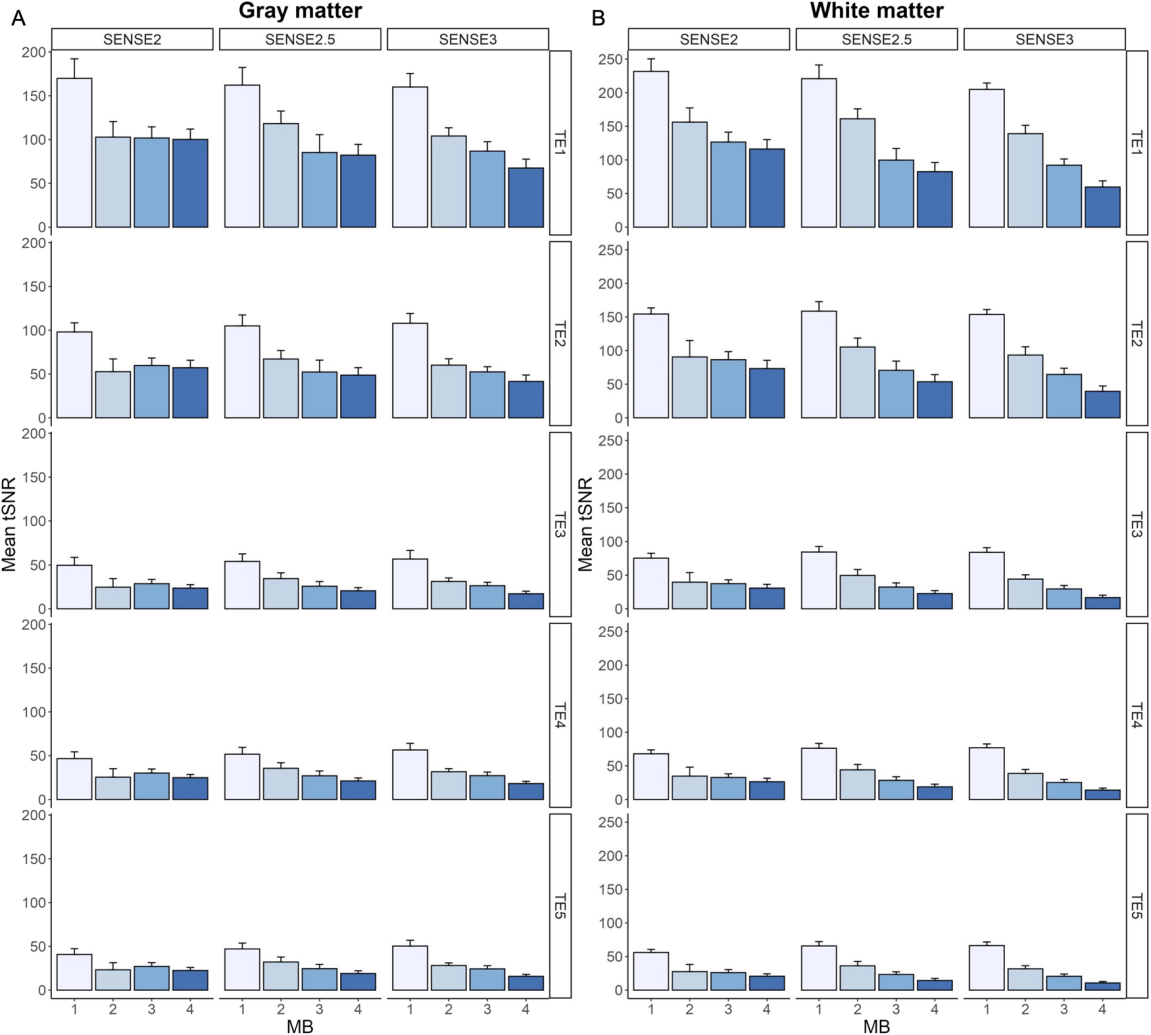
Group-level mean tSNR across acceleration factor combinations for TE_1-5_in gray (A) and white matter (B). There were no significant differences in tSNR in gray or white matter across TEs between protocols with feasible acquisition parameters (i.e., full brain coverage ≥120 mm, TE_1_< 10 ms, TE_5_< 100 ms).

**Fig. 4.**
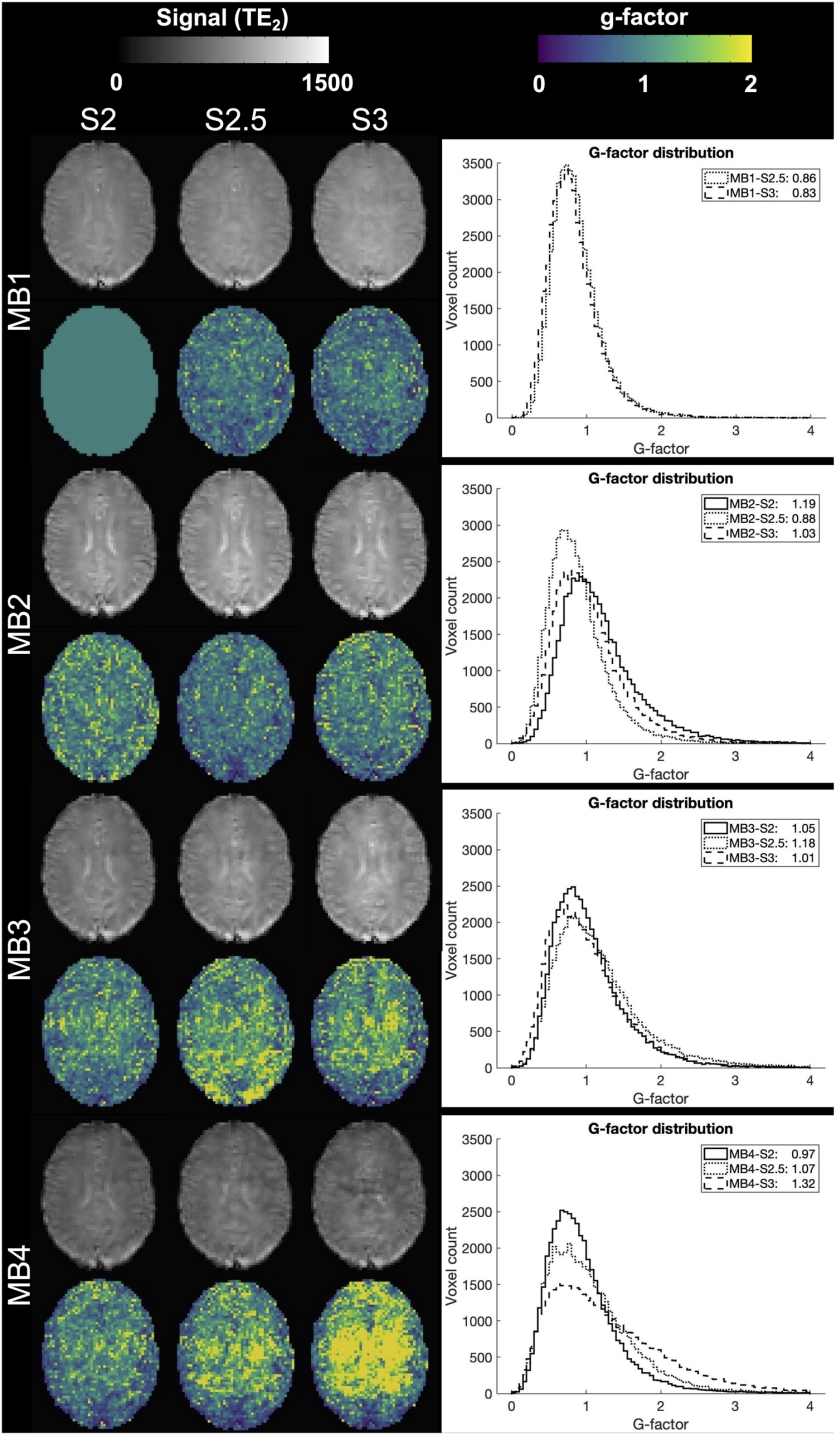
TE_2_per acquisition and corresponding g-factor analysis in a representative subject. Increasing g-factor values may correspond to acceleration-associated artifacts seen in TE_2_. Histograms exhibiting the distribution of whole-brain voxelwise g-factor values are provided.

Figure 3shows mean tSNR trends in gray matter and white matter across echoes and acceleration factors; specific values are provided in Supplementary MaterialsTable S2. Gray matter tSNR was lower than that in white matter as expected, with decreasing tSNR observed across echoes and with increasing acceleration. Post-hoc comparisons are reported in Supplementary MaterialsTable S3(*p *< 0.05, corrected for multiple comparisons). While not significant for all protocols, the MB2-SENSE2.5 acquisition showed the highest tSNR values from this group. This was further supported by a quantitative assessment of artifacts due to acceleration via g-factor, calculated for each accelerated protocol on TE_2_(Fig. 4). Quantitative assessment of T_2_^*^and T_2_values in gray and white matter ROIs revealed no significant differences between any of the MB2 and MB3 combinations across SENSE factors (*p *< 0.05, corrected for multiple comparisons, Supplementary MaterialsFig. S2). Based on these analyses, the MB2-SENSE2.5 combination was selected for implementation in task-based fMRI due to its minimal loss in tSNR and sufficient improvement in spatial and temporal resolution.

### 3.2 SAGE-fMRI: Validation

**Fig. 5.**
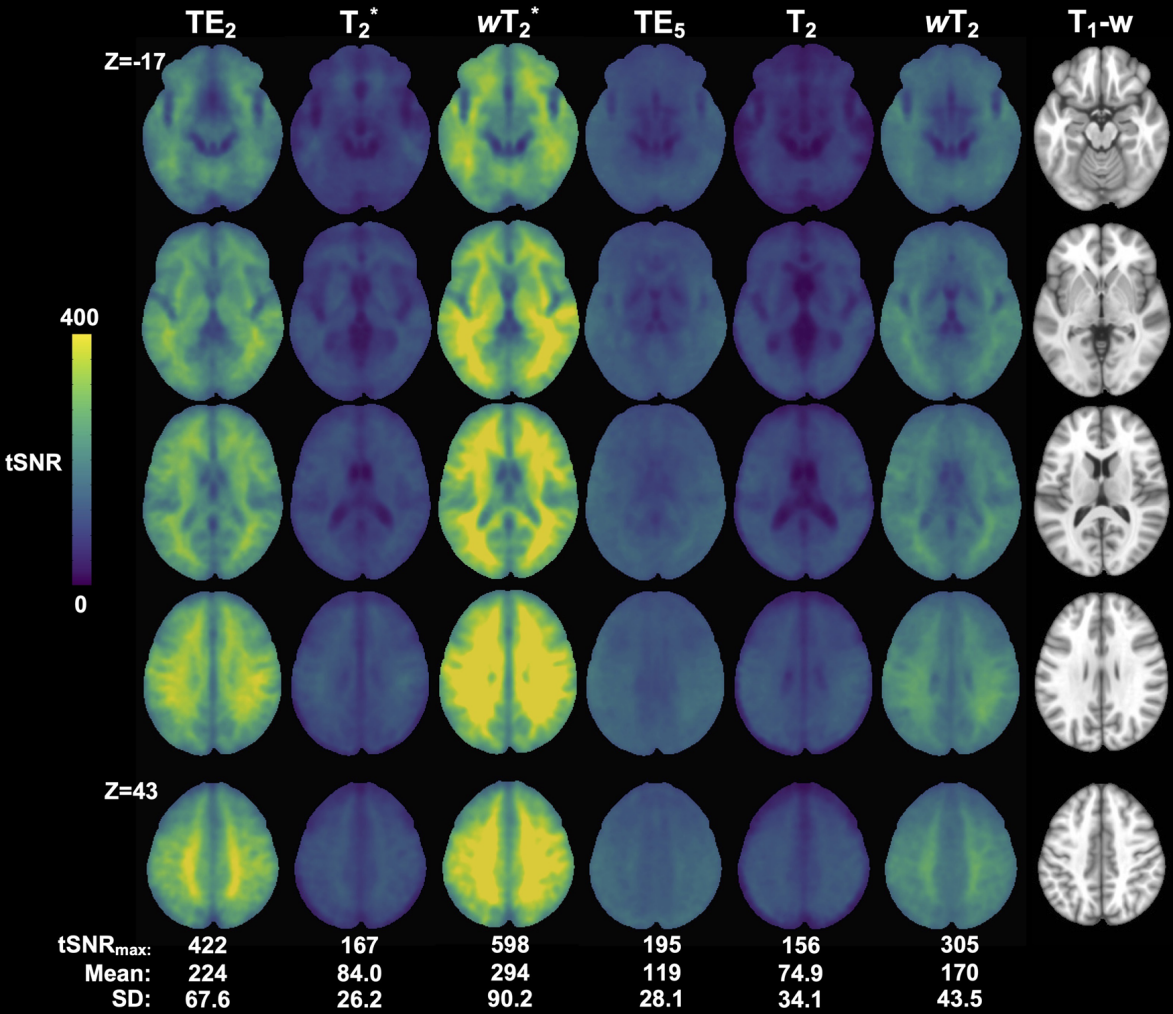
Group-level tSNR maps for the working memory task across macro- (TE_2_, SAGE T_2_*, and SAGE*w*T_2_^*^) and microvascular (TE_5_, SAGE T_2_, and SAGE*w*T_2_) acquisitions. Anatomical T_1_-w image is included for reference.

Weighted SAGE maps had the highest tSNR values in raw group average maps within their respective vascular scales (Fig. 5; Supplementary MaterialsTable S4). For both T_2_^*^- (i.e., T_2_^*^,*w*T_2_^*^, and TE_2_) and T_2_-dominant (i.e., T_2_,*w*T_2_, and TE_5_) analyses, weighted SAGE maps showed significantly higher tSNR across the brain compared to quantitative SAGE maps (*w*T_2_^*^> T_2_^*^: maximum*t*-score (*t*_max_) = 43.1 significant for 99.5% total brain volume;*w*T_2_> T_2_:*t*_max_= 24.8, significant for 99.3% total brain volume) and single-echo fMRI (*w*T_2_^*^> TE_2_:*t*_max_= 17.1, significant for 43.5% total brain volume;*w*T_2_> TE_5_:*t*_max_= 28.9, significant for 70.0% total brain volume). Notably, in the temporal and inferior frontal lobes, there was no significant difference between T_2_^*^and TE_2_, and in portions of these regions, T_2_^*^tSNR was significantly higher than TE_2_(*t*_max_= 4.7, significant for 1.1% of temporal and inferior frontal lobe volumes). This was not seen between T_2_and TE_5_except in some inferior frontal lobe white matter; brain regions in which T_2_were significantly higher than TE_5_were in the thalamus and basal ganglia (*t*_max_= 3.6, significant for 1.49% of thalamus and basal ganglia volumes).

**Fig. 6.**
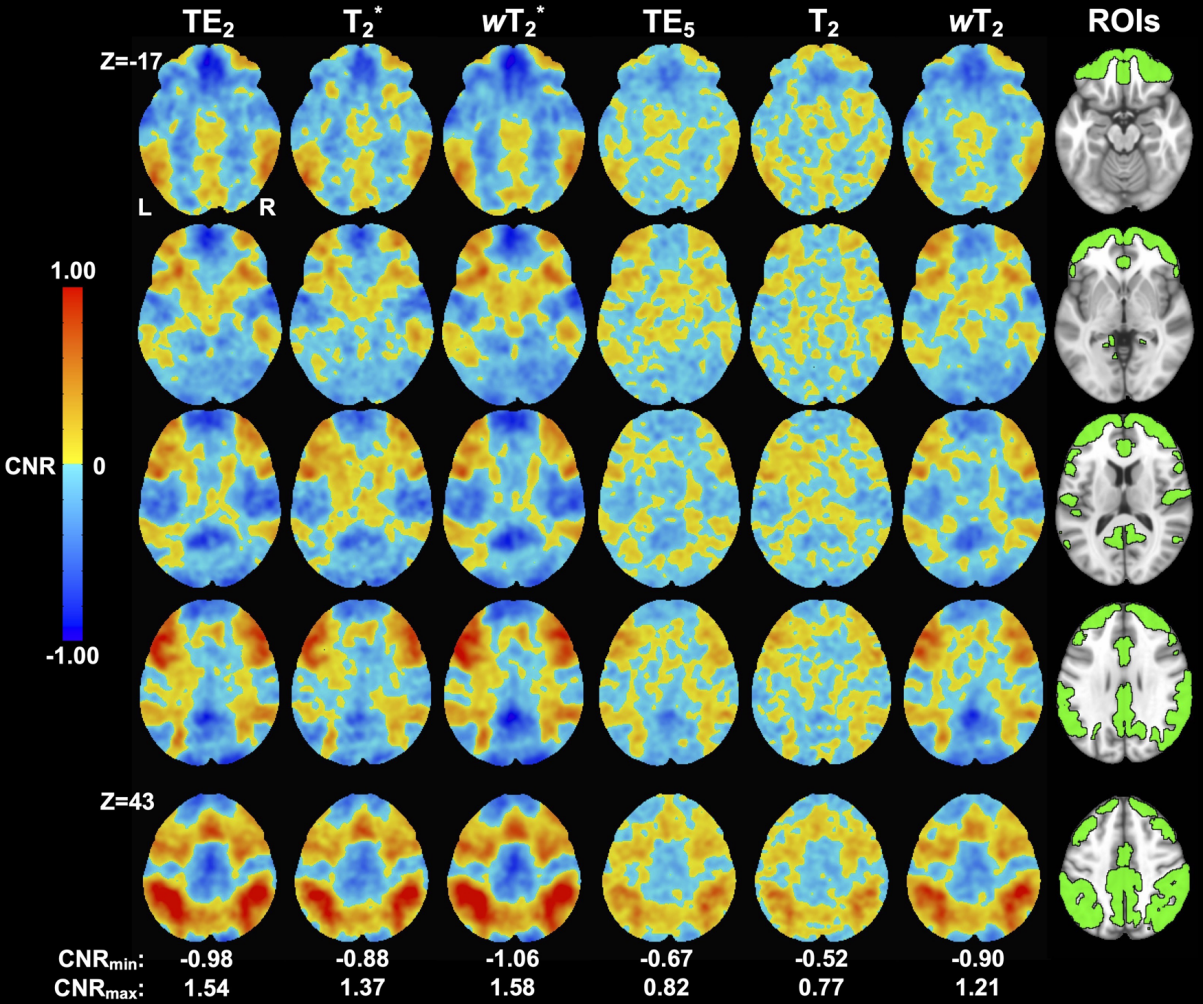
Group-level CNR maps for the working memory task across macro- (TE_2_, SAGE T_2_*, and SAGE*w*T_2_^*^) and microvascular (TE_5_, SAGE T_2_, and SAGE*w*T_2_) acquisitions. Anatomical T_1_-w image with task-related regions of interest (ROIs) is included for reference.

CNR values were slightly higher for weighted SAGE than quantitative SAGE and single-echo methods, respectively (Fig. 6; Supplementary MaterialsTable S4). One of the more notable gains occurred in the inferior frontal gyrus, where higher CNR averages and maximum values are found in the weighted SAGE analyses. This is further supported by the*t*-scores.

**Fig. 7.**
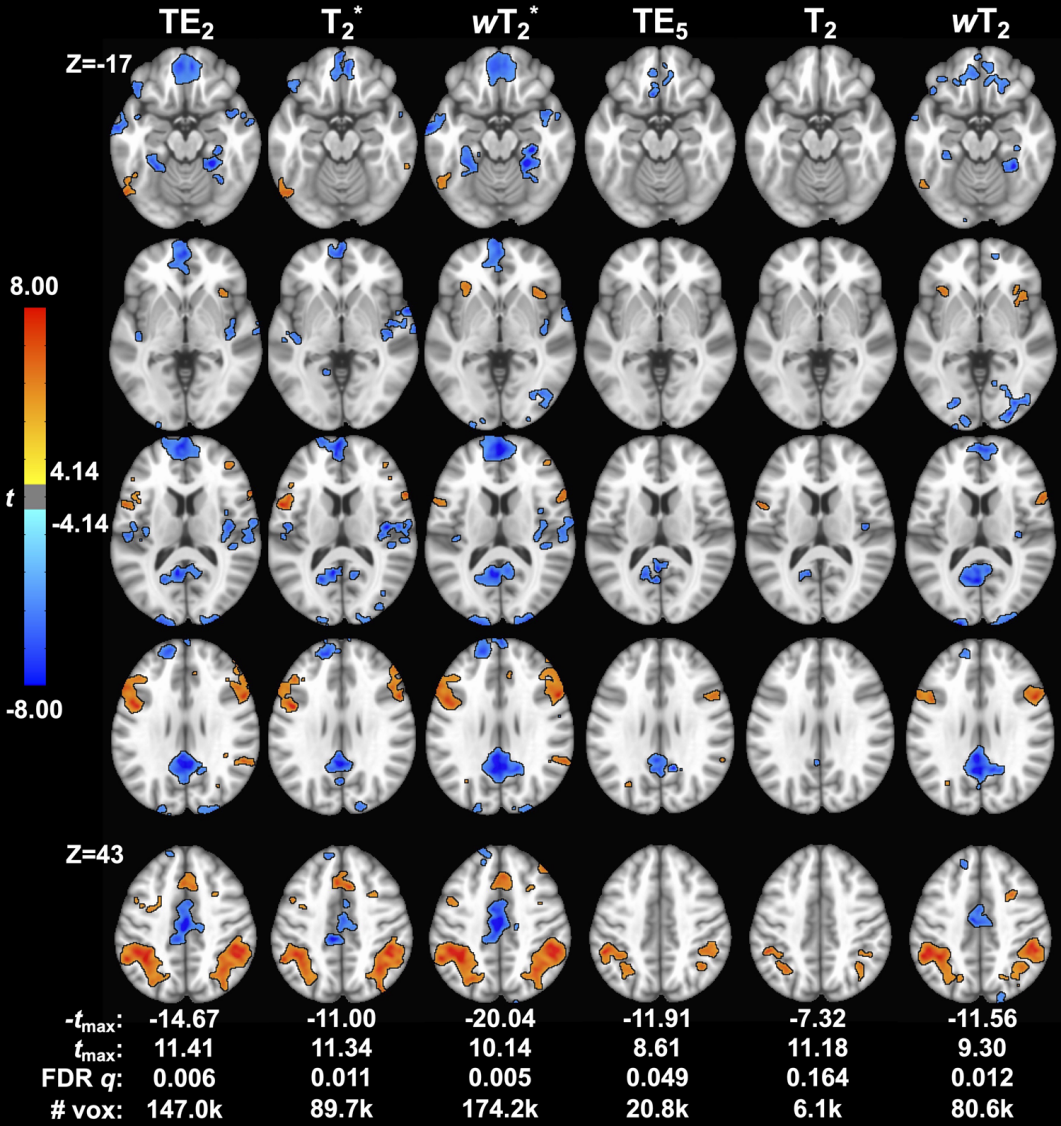
Group-level*t*-maps for the working memory task across macro- (TE_2_, SAGE T_2_*, and SAGE*w*T_2_^*^) and microvascular (TE_5_, SAGE T_2_, and SAGE*w*T_2_) acquisitions. # vox = voxel count for significant functional activation.

**Fig. 8.**
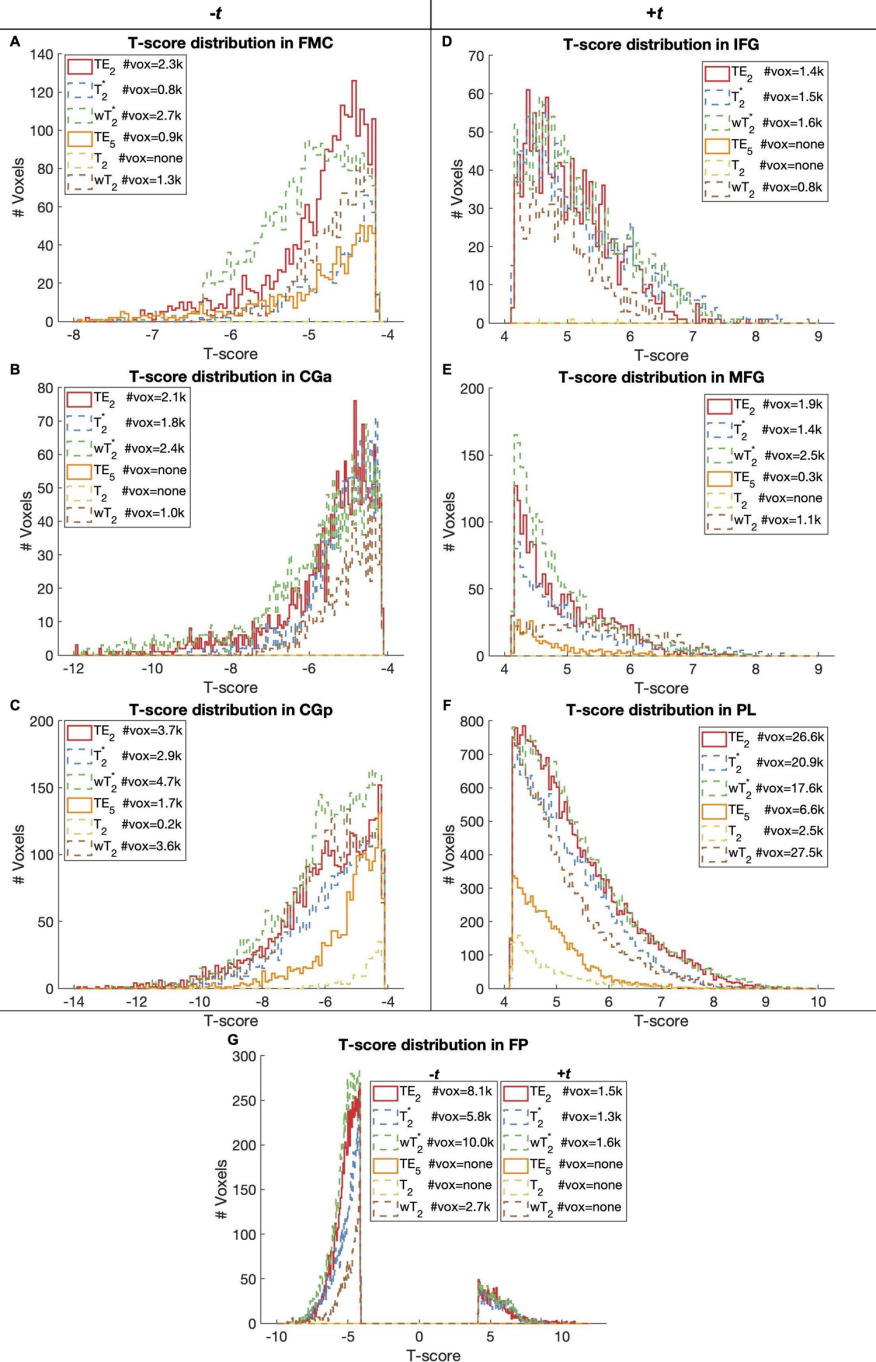
*N*-back task-based activation histograms for ROIs with negative (A-C; G) and positive (D-G) activation. Each histogram shows the distribution of statistically significant voxels (*p *< 0.001, cluster size corrected) for each single-echo or SAGE-based method, with the significant voxel count in the legend. FP had both positive (dorsolateral prefrontal cortex) and negative (dorsomedial prefrontal cortex) activation. Voxel clusters of less than 100 were removed for visualization. FMC = Frontal Medial Cortex; CGa = Cingulate Gyrus anterior division; CGp = Cingulate Gyrus posterior division; IFG = Inferior Frontal Gyrus; MFG = Middle Frontal Gyrus; PL = Parietal Lobe; FP = Frontal Pole.

For the working memory task, there was significant activation (*p *< 0.001, cluster size corrected) in ROIs across T_2_^*^- and T_2_-dominant maps for single-echo and SAGE methods (Fig. 7; Supplementary MaterialsTable S4); activation and tSNR for the remaining SAGE echoes are provided in Supplementary MaterialsFigure S3. T_2_^*^-dominant maps showed similar activation and deactivation; SAGE*w*T_2_^*^showed more robust deactivation (-*t*_max_) and a larger voxel count for significant activation and deactivation than TE_2_and T_2_^*^methods. T_2_-dominant maps showed similar deactivation, except for*w*T_2_maps revealing significant deactivation in the anterior cingulate gyrus and frontal pole where TE_5_and T_2_did not (Fig. 8; Supplementary MaterialsTable S4). Both SAGE T_2_and*w*T_2_map had higher*t*_max_than TE_5_; the*w*T_2_map also had a higher voxel count for significant activation than other SE-based methods. As noted with CNR, there were noticeable gains in significant voxels from TE_2_to SAGE*w*T_2_^*^, where the former showed unilateral activation in the right inferior frontal gyrus and the latter showed bilateral activation; bilateral activation was also seen for*w*T_2_, where neither TE_5_nor T_2_showed significant activation in this region. Notably, there is an increase in deactivated voxels in the frontal medial cortex for weighted SAGE analyses compared to single-echo counterparts, likely reflecting an improvement in sensitivity and (for GRE) susceptibility-induced dropout in this region.

A preliminary comparison between SAGE-fMRI (TE_2_and TE_5_) and separate single-echo BOLD acquisitions optimized for a single contrast (both GRE and SE) is shown in a representative subject in Supplementary MaterialsFigure S4. In the single-echo GRE acquisition, the impact of both the slight difference in TE (27 ms for SAGE-TE_2_vs. 30 ms for GRE) and the much shorter TR for GRE is assessed. Minimal differences are observed between the SAGE-TE_2_and GRE with an equal number of dynamics (i.e., GRE_alt_dyn_), suggesting that the impact of TE is minimal. However, comparisons between SAGE-TE_2_and the full (i.e., 225 dynamics) GRE analysis demonstrate the benefits of more volumes enabled by the shorter TR. The optimized SE shows more significant voxels compared to SAGE-TE_5_, which may be the result of the shorter TE (80 ms vs. 97 ms, respectively). It should be noted that the SAGE-TE_4_has a similar TE (78 ms) to the optimized SE protocol, and the subsequent results for SAGE-TE_4_more closely matched that of the single-echo SE (data not shown).

Effect size analysis showed similar trends as the*t*-test results (Supplementary MaterialsFig. S5). SAGE*w*T_2_^*^showed a larger maximum effect size for deactivation (-*g*_max_) and a larger count for voxels with at least a large effect size compared to TE_2_and T_2_^*^methods. To a greater extent, SAGE*w*T_2_had higher counts for voxels with at least a large effect size than TE_5_or T_2_, but the maximum effect sizes were more similar across T_2_-dominant maps. There was a large effect cluster in SAGE*w*T_2_posterior cingulate cortex, as expected and seen in T_2_^*^-dominant maps, that was not found in TE_5_or T_2_methods.

For the visual task, there was significant activation (*p *< 0.001, cluster size corrected) in ROIs across single-echo and SAGE methods (Supplementary MaterialsFig. S6). SAGE*w*T_2_maps showed significant activation in the temporal occipital fusiform and superior lateral occipital cortices, whereas TE_5_and T_2_did not. Both SAGE*w*T_2_^*^and*w*T_2_maps had higher*t*_max_and a higher voxel count for significant activation than other T_2_^*^- and T_2_-dominant methods, respectively.

Effect size analysis for the visual task showed that SAGE*w*T_2_^*^and*w*T_2_had higher maximum effect sizes and counts for voxels with at least a large effect size than their single-echo (TE_2_and TE_5_) and quantitative (T_2_^*^and T_2_) counterparts (Supplementary MaterialsFig. S7).

**Fig. 9.**
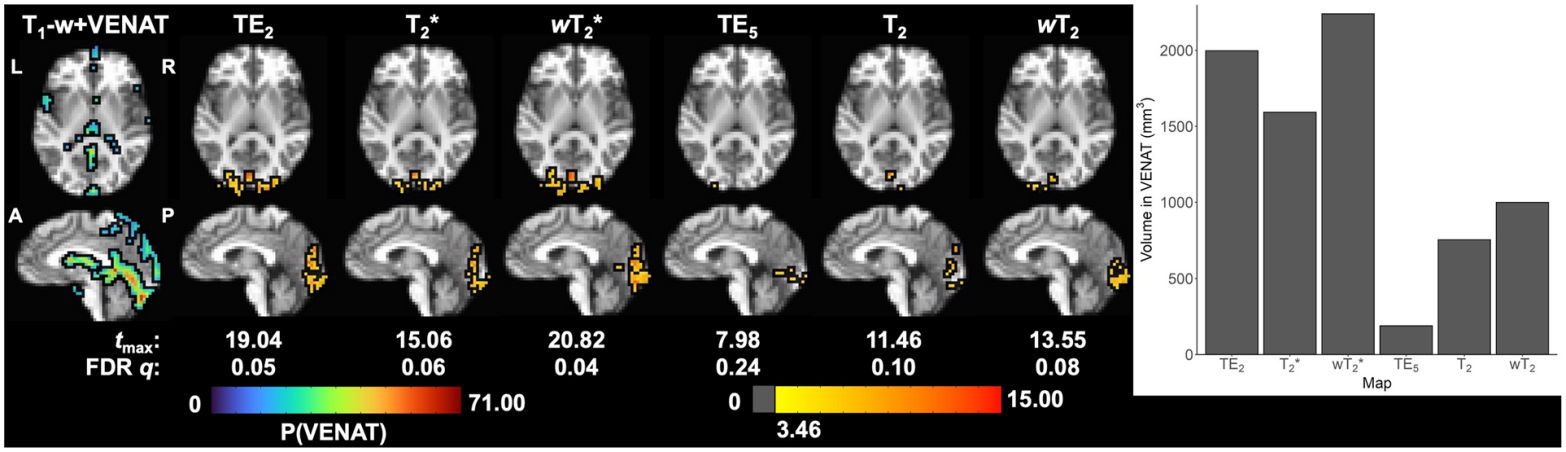
Task-based activation (corrected for multiple comparisons, FDR*q*< 0.05) in a representative subject for all SAGE macro- (TE_2_, SAGE T_2_*, and SAGE*w*T_2_^*^) and microvascular (TE_5_, SAGE T_2_, and SAGE*w*T_2_) analyses. The VENAT probabilistic atlas, co-registered into native space, is provided for reference corresponding to large draining veins in the brain. VENAT and activation maps are overlaid on T_1_-w anatomical image co-registered into native space. Macrovascular-weighted activations overlap with the straight, transverse, and sagittal sinuses, whereas microvascular-weighted activations appear to be largely constrained to gray matter of the visual cortex. P(VENAT) = VENAT atlas probability estimate.

Activation for the visual task was observed in the occipital lobe for both GRE (i.e., TE_2_) and SE (i.e., TE_5_) fMRI, as well as multi-echo SAGE-fMRI (FDR correction for multiple comparisons,*q *< 0.05;Fig. 9). When referencing the VENAT atlas, there is significant task-based functional activation that appears to overlap with the draining straight and transverse sinuses in the GRE-based analyses. Conversely, SE-based functional activation appears to be largely localized within the gray matter of the visual cortex.

## 4 Discussion

In this study, we implemented and optimized a multi-echo and multi-contrast fMRI method based on the SAGE sequence, with proposed fMRI weighting strategies to combine the advantages of MGE- and SE-fMRI. MGE-fMRI methods have several benefits over single GRE EPI, most notably in reduced image artifacts and improved BOLD sensitivity (Cohen et al., 2018;Evans et al., 2015;Kundu et al., 2013;Poser et al., 2006;Posse et al., 1999;Speck & Hennig, 1998). Adding an additional echo with a shorter TE has virtually no effect on any other pulse sequence parameter: the typical BOLD signal (TE_2_) is preserved, the spatial resolution and slice coverage are maintained, and the temporal resolution and acquisition time are unchanged. With the inclusion of a shorter first TE, the BOLD sensitivity was previously shown to improve by a minimum of 11%, with some sensitivity gains reported up to 67% in regions with high susceptibility-induced signal dropouts (Poser et al., 2006). Other studies have demonstrated that MGE-BOLD improves denoising, yielding higher SNR for functional connectivity (Cohen, Yang, et al., 2021;Kundu et al., 2013) and task-based activation analysis (Cohen, Jagra, et al., 2021;Cohen et al., 2018). However, multi-echo methods suffer many of the same limitations as single GRE methods: sensitivity to large vessels and some (albeit improved) regional signal dropout due to susceptibility. Presently, we observed that GRE-fMRI functional activation analyses may be influenced by sensitivity to large draining veins within the context of a visual task (Fig. 9), in agreement with what we expect based on the literature concerning vessel size sensitivities (Boxerman et al., 1995;Weisskoff et al., 1994).

SE methods have much lower BOLD sensitivity but are not sensitive to susceptibility-induced signal drop-out, and thus these methods have advantages in artifact-prone regions such as the inferior frontal lobe and temporal lobes. An additional benefit of SE-based contrast is improved specificity to the microvasculature (Boxerman et al., 1995;Stables et al., 1998;Weisskoff et al., 1994), due to reduced sensitivity to large vessels. At clinical field strengths (such as 3T), SE-fMRI comprises both intravascular (blood) T_2_effects and extravascular (microvessel) effects; this typically necessitates either the use of longer TEs (Ragot & Chen, 2019) or higher field strengths (Duong et al., 2003;Lee et al., 1999) to minimize intravascular effects. Although SE contrast provides higher microvascular specificity (Parkes et al., 2005), this comes at a cost of BOLD sensitivity (Duong et al., 2003;Lowe et al., 2000;Parkes et al., 2005). Other approaches to overcome this limitation could include the use of ultra-high-resolution GRE-fMRI; notably, laminar fMRI has previously shown activation localized within gray matter with sub-millimeter resolution at 3T (Knudsen et al., 2023). In the present study, SE-fMRI showed lower BOLD sensitivity (i.e., smaller volume of clusters with task-based activation), but perhaps higher spatial specificity (i.e., less sensitivity to large draining veins), as activation is mostly within the visual cortex (Fig. 9). Finally, SE-BOLD is less sensitive to physiological noise (Ragot & Chen, 2019) which is a known confounder for fMRI analysis (Birn et al., 2006;Chang et al., 2009). Multi-contrast SAGE-fMRI combines the strengths of MGE- and SE-fMRI.

Another potential advantage for SAGE-fMRI is the direct quantification of dynamic R_2_^*^and R_2_time courses. Although quantitative T_2_^*^and T_2_are more directly related to changes in BOLD (Buxton, 2013;Poser et al., 2006;Speck & Hennig, 1998), previous research has shown that T_2_^*^is less robust compared to weighted signal combinations (Posse et al., 1999) that aim to maximize BOLD contrast sensitivity by considering local T_2_^*^values (Poser et al., 2006). In this study, we observed smaller clusters of significant task-based activation and effect size for both T_2_^*^and T_2_relative to*w*T_2_^*^and*w*T_2_, respectively; by weighting for T_2_^*^and T_2_, higher BOLD contrast sensitivity is expected (i.e., CNR), yielding larger clusters. The T_2_^*^and T_2_clusters were also smaller than any of the single-echo fMRI clusters. Smaller T_2_^*^clusters could be due to separation of BOLD and non-BOLD signal that comes with multi-echo fMRI, possibly improving specificity to the BOLD effect compared to single GRE (Kundu et al., 2012). Smaller SE clusters likely result from both reduced BOLD contrast and from higher spatial specificity (from reduced sensitivity to draining veins) (Duong et al., 2003;Lowe et al., 2000;Parkes et al., 2005). The former is a disadvantage for SE-fMRI, while the latter may be advantageous. In this study, we consistently observed the largest gains across metrics from TE_5_and T_2_to*w*T_2_; by weighting for T_2_, there is a tradeoff between the loss in microvascular specificity and the gain in BOLD sensitivity. However, as all contrasts can empirically be compared using SAGE-fMRI, we anticipate that the higher spatial specificity associated with TE_5_and T_2_contrast can provide insight into the underlying sources of activation clusters observed with*w*T_2_contrast.

The SAGE sequence includes multiple echoes and multiple types of contrast, but a drawback of this technique is the necessary trade-offs between sequence parameters. In various iterations of this sequence, GRAPPA, SENSE, PF, and MB methods have been added to accelerate image acquisition (Han et al., 2021;Manhard et al., 2019,^2021^;Skinner et al., 2014), yielding better spatial and temporal resolution. Studies have also shown that removing the ASE readouts may enable shorter SE TEs, an approach entitled simplified SAGE (Stokes & Quarles, 2016;Stokes, Skinner, et al., 2016). In this study, various SENSE and MB factors were tested with standard five-echo SAGE, and the requisite parameters included 3 mm isotropic resolution, whole-brain coverage (≥120 mm), TE_1_less than 10 ms, TE_5_less than 100 ms, and TR equal to 3 s. The TE requirements were in place to ensure adequate signal in each echo. Studies requiring higher spatial resolution would have tradeoffs with TEs, and thus slice coverage may diminish. Conventional GRE-based EPI can typically achieve shorter TRs (on the order of 2 s or less) with whole-brain coverage, but the inclusion of SE contrast necessitates the longer TR for SAGE. In theory, steady-state effects (T_1_-recovery) decrease the signal intensity at shorter TRs; on the other hand, a longer TR decreases the number of image volumes that can be acquired in a fixed scan length. In general, shorter TRs can give higher detection sensitivity (for a fixed scan time), ignoring serial correlation effects (Sahib et al., 2016). This can be readily seen in the direct comparison between SAGE-fMRI and single-echo GRE, where the shorter TR for the latter enabled improved sensitivity due to the increased number of dynamics. In this study, we held the SAGE-fMRI TR constant at 3 s, although it could have been shortened with increasing acceleration. While the SAGE-fMRI acquisition was optimized here for our specific spatiotemporal requirements, it is important to note that future implementations could further optimize SAGE-fMRI to best address different research questions. Furthermore, other approaches such as multi-shot EPI or EPI with keyhole could be explored to modulate spatial or temporal resolution as needed (Küppers et al., 2022;Wang et al., 2019).

Presently, we have proposed optimized fMRI weighting strategies for SAGE-fMRI that leverage the multiple echoes and multiple types of contrast. Few studies have considered SAGE for fMRI. For example, SAGE-fMRI was previously performed in a single subject using a breath-hold stimulus, demonstrating the feasibility of this method (Schmiedeskamp et al., 2014). Additionally, SAGE-fMRI has been proposed for assessing relative vessel sizes during functional activation (Newton et al., 2014), similar to vessel size imaging methods for perfusion MRI (Kiselev et al., 2005).Glielmi et al. (2010)previously compared simultaneous arterial spin labeling (ASL)-fMRI, GRE-BOLD, and SE-BOLD using a dual-GRE and SE method, similar to simplified SAGE implementations that exclude ASE acquisitions (Stokes & Quarles, 2016;Stokes, Skinner, et al., 2016); more specifically, ASL-based measures of cerebral blood flow (CBF) were obtained from tag-control difference images of the first echo, while GRE-BOLD and SE-BOLD were obtained from averaged images (tag-control) of the second GRE and the SE, respectively. Using a visual stimulus at 3T, they showed that ASL-CBF was the most localized and had the highest specificity, but the lowest sensitivity. GRE-BOLD had the largest activation and the highest sensitivity, while SE-BOLD was a compromise between ASL-CBF and GRE-BOLD (that is, more localized than GRE-BOLD and more sensitive than ASL-CBF) (Glielmi et al., 2010). However, the present study is the first to propose an optimized analysis for SAGE-fMRI based on echo-weighting strategies that leverage the multiple echoes and types of contrast simultaneously.

Comparing SAGE-based T_2_^*^-weighted combined images to MGE-fMRI was beyond the scope of the present work but could be an important future direction for SAGE-fMRI. Additionally, while two GREs are implemented in SAGE-fMRI, other studies have utilized three to five to fit for T_2_^*^(Cohen et al., 2018;Evans et al., 2015;Kundu et al., 2013;Poser et al., 2006;Posse et al., 1999;Speck & Hennig, 1998). Because T_2_^*^values vary across the brain (Hagberg et al., 2002), and gray matter signal decay behaves monoexponentially over a range of TEs (Hagberg et al., 2002;Speck & Hennig, 1998), increasing the number of GREs can enable more accurate estimations of T_2_^*^. Moreover, increasing acceleration can reduce EPI readouts, which may enable more optimal TE choices to maximize T_2_^*^contrast in various brain regions (Newbould et al., 2007).The range of TEs (∆TE) may also be important to the accurate estimation of T_2_^*^; ∆TE should be approximately equal to or greater than the true T_2_^*^(Shin et al., 2023). Presently, the SAGE-fMRI acquisition had ∆TE of 19 ms; both the number of echoes and ∆TE are somewhat mitigated by the inclusion of two mixed-contrast ASEs (four echoes, ∆TE = 70 ms). Empirical examination of the difference in T_2_^*^estimation between SAGE-fMRI and the more typical multi-GRE approach may be important for understanding the effect of number and contrast of echoes on T_2_^*^estimation. Despite this, quantitative T_2_^*^values from the optimized SAGE protocol were comparable to those reported in literature for other multi-echo, multi-contrast acquisitions (Küppers et al., 2022), while SAGE-T_2_was slightly overestimated in gray matter (Supplementary MaterialsTable S1). This could be due to differences in acquisitions related to minimum TE achievable or only acquiring one true SE signal with SAGE.

Herein, we have demonstrated that SAGE can be used in fMRI for whole-brain, task-based multi-echo analyses. SAGE-fMRI offers many potential applications, such as resting-state functional connectivity. Because SAGE-fMRI offers improvements and alternative contrasts to those susceptible to signal dropout, it may be applied in pathologies localized within the medial temporal and inferior frontal lobes, such as Alzheimer’s disease and frontotemporal dementia. Moreover, SAGE-fMRI may be of particular interest when pathology is localized in the microvasculature. Additionally, SAGE-fMRI can be used to quantify multiple metrics such as oxygen extraction fraction and cerebrovascular reactivity, thus providing a more comprehensive picture of vascular and metabolic function in the brain on complementary vascular scales.

There are several important limitations to this study and more generally to SAGE-based fMRI. Limitations regarding the optimization study include that only 40 dynamics were acquired and TEs varied across protocols, which could affect tSNR estimation; additionally, g-factor calculations are reported relative to the least accelerated protocol rather than to a non-accelerated protocol. Another limitation is related to the relaxation-weighting factors implemented herein. By including ASE signals with mixed sensitivities and a SE at 3T (where intravascular contributions remain), it is important to acknowledge that the T_2_-weighted analyses likely include T_2_^*^contributions. Additionally, the proposed weighting strategy inherently necessitates analysis with two signals (*w*T_2_^*^and*w*T_2_), whereas future studies may prefer a single optimized signal for analysis. Other options to reduce data dimensionality include a simple summation (Posse et al., 1999), CNR-weighting (Poser et al., 2006), vessel-size weighting (Kiselev et al., 2005;Newton et al., 2014;Troprès et al., 2001), or even other relaxation-weightings, such as T_2_’. Further research should investigate the advantages of these methods in the context of combined contrast across GRE, ASE, and SE readouts. An additional challenge to SAGE-fMRI, specifically fitting for dynamic T_2_^*^and T_2_values, is the relatively long time required for fitting. This was presently addressed by implementation of a linear least-squares fitting solution (Sisco et al., 2022). Moreover, we previously showed that a simplified SAGE approach may enable real-time quantification of relative T_2_^*^and T_2_changes (Stokes & Quarles, 2016;Stokes, Skinner, et al., 2016), which may be similarly amenable for SAGE-fMRI.

Finally, one of the challenges for fMRI is the complex origin of the BOLD signal, which depends on physiological and biophysical effects, as well as non-BOLD factors such as pulse sequence parameters (Buxton, 2013). We hypothesize that the inclusion of multiple types of contrast could enable separation of intravascular and extravascular effects at clinical field strengths, though such an investigation is outside the scope of this study. Vessel size specificity may also be attained by tuning the combination of GRE and SE signals (Newton et al., 2014). Moreover, SAGE-fMRI may permit more advanced analysis into spatiotemporal differences between GRE and SE activation, which are critical for understanding neurovascular dynamics in a range of pathologies. A more detailed characterization and quantification of BOLD signal contributions within a single experiment, using the SAGE-fMRI framework developed in this study, may improve our understanding of fMRI mechanisms and interpretation of the origins of functional activation.

## 5 Conclusions

SAGE-fMRI enables the simultaneous acquisition of multiple echoes and contrasts, with a wide range of possible fMRI applications. Using SAGE-fMRI, two contrast mechanisms can be feasibly acquired, separated, and enhanced with the relaxation-weighted summation analysis methods. SAGE-fMRI couples the high BOLD sensitivity from MGE acquisitions with the potential microvascular specificity from SE acquisitions. SAGE-fMRI provides several advantages, including improving tSNR and BOLD CNR for more accurate analysis. This combination of multiple echoes and contrast mechanisms bolsters the confidence in the resulting functional maps. SAGE-fMRI may ultimately provide new insight into the complex contributions to BOLD signal and improve spatial localization of brain activation. Overall, SAGE-fMRI provides flexibility for analysis and can be optimized for different contrast mechanisms.

## Supplementary Material

Supplementary Material

## Data Availability

Data and code may be made available upon the construction of a formal data sharing agreement.
