# Supplementary material for: Optimization and validation of multi-echo, multi-contrast SAGE acquisition in fMRI
Source: Imaging Neurosci (Camb). 2024 Jul 2;2:imag-2-00217. doi: 10.1162/imag_a_00217 (PMC11497078; doi:10.1162/imag_a_00217)
Supplement: Supplementary Material [file imag_a_00217-supp.pdf]

Supplementary Materials

Figure S1. SAGE pulse sequence (A). Weighting factors for each SAGE signal (5 echoes, S_1-S_5) for the T_2^*- and T_2-weighted analyses, respectively (B). The relaxation-weights vary both spatially and across echoes, based on their derived contribution to BOLD contrast (see Eqs. 2 and 3).

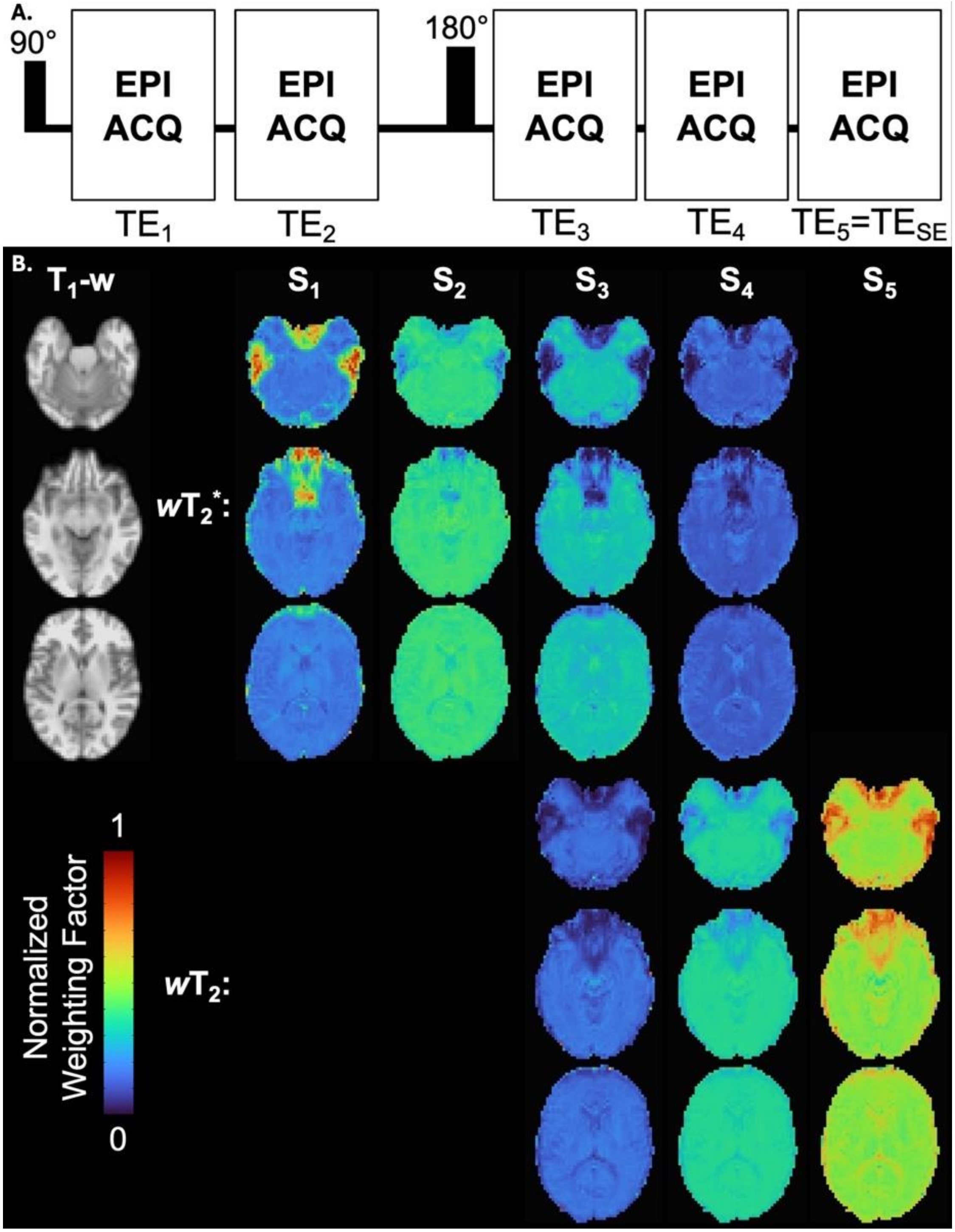

Figure S2. ROIs for optimization T_2^* and T_2 fits in standard space template (A). Group-level mean and standard deviation for T_2^* (B-E) and T_2 (F-I) across acceleration factor combinations.

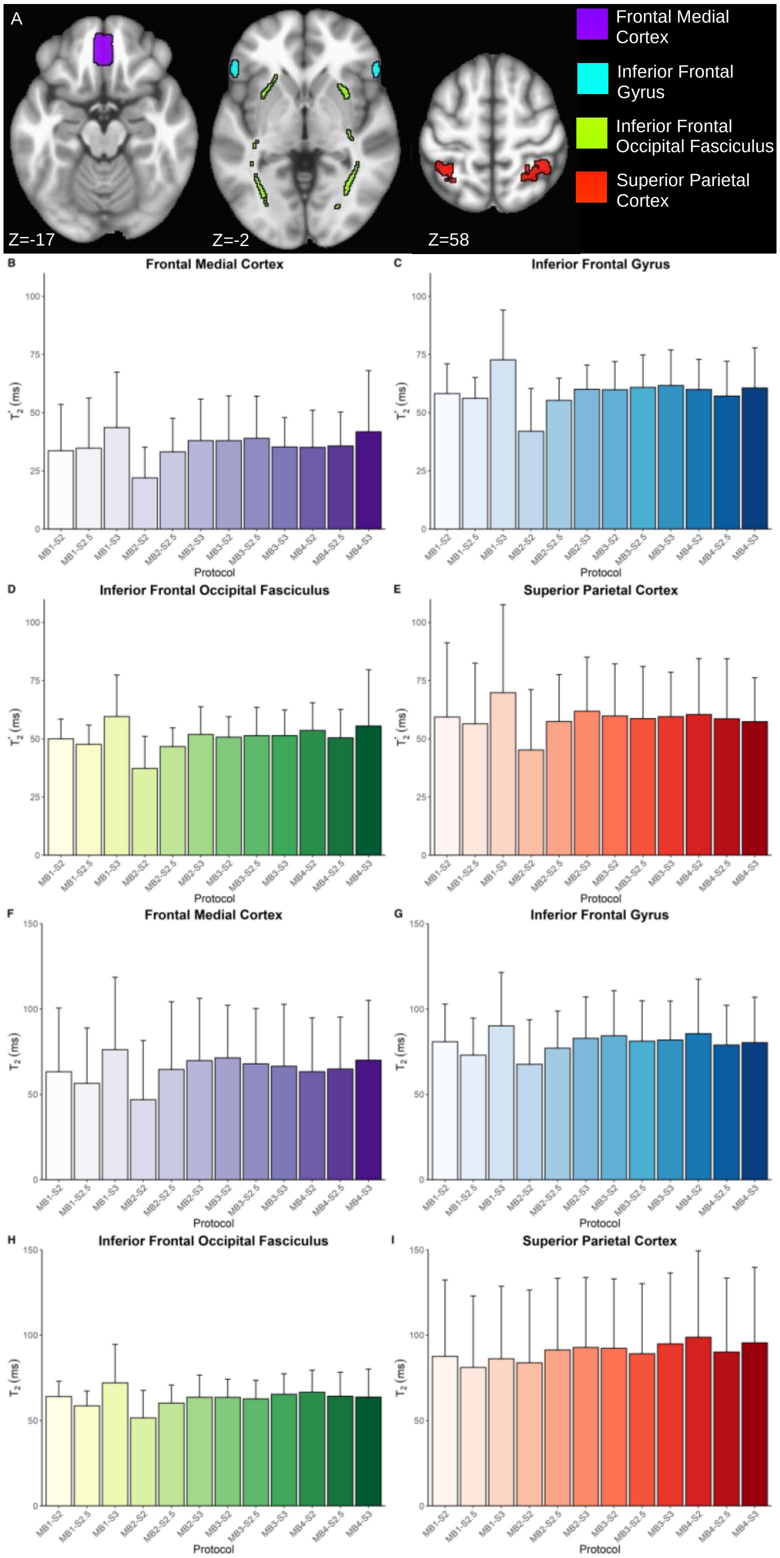

Figure S3. Group-level *t*-maps (A) and tSNR (B) for the working memory task for SAGE TE_1, TE_3, and TE_4 acquisitions. # vox = voxel count for significant functional activation. The corresponding results for TE_2, TE_5, and multi-echo combinations are reported in Figures 5 and 6 for comparison.

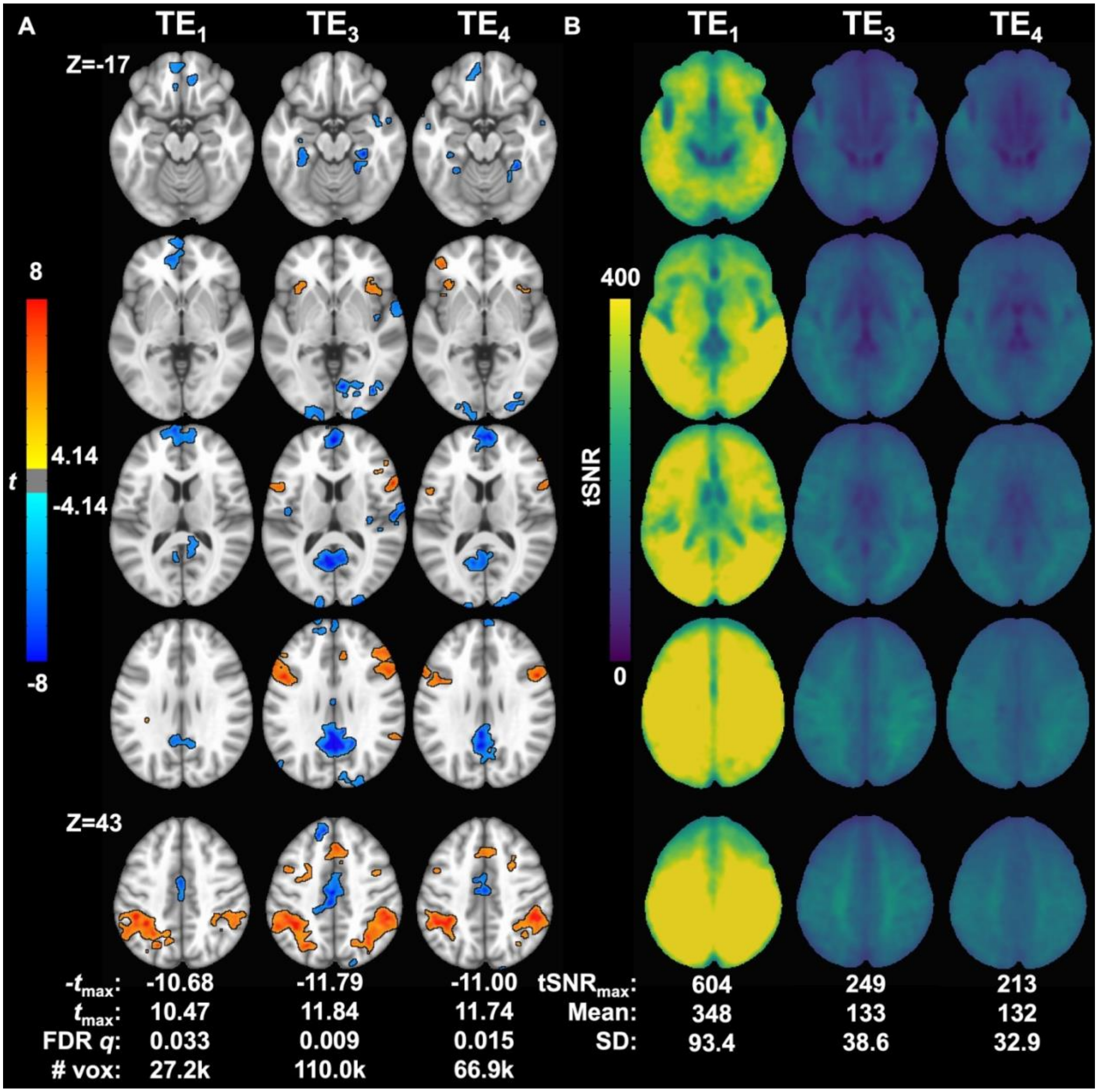

Figure S4. Task-based activation (p<0.001, cluster size corrected) in a representative subject for the *N*-back working memory task for separate SAGE, GRE, and SE acquisitions. To illustrate that number of dynamics may be driving differences between SAGE-TE_2 and GRE, GRE_{alt_dyn} shows the results when using the same number of dynamics as SAGE echoes and the SE, by including every other dynamic in the analysis. # dyn = number of dynamic volumes; # vox = number of voxels with significant activation; % vox_{ROI} = percentage of total voxels showing significant activation that fall within well-established ROIs for the *N*-back task.

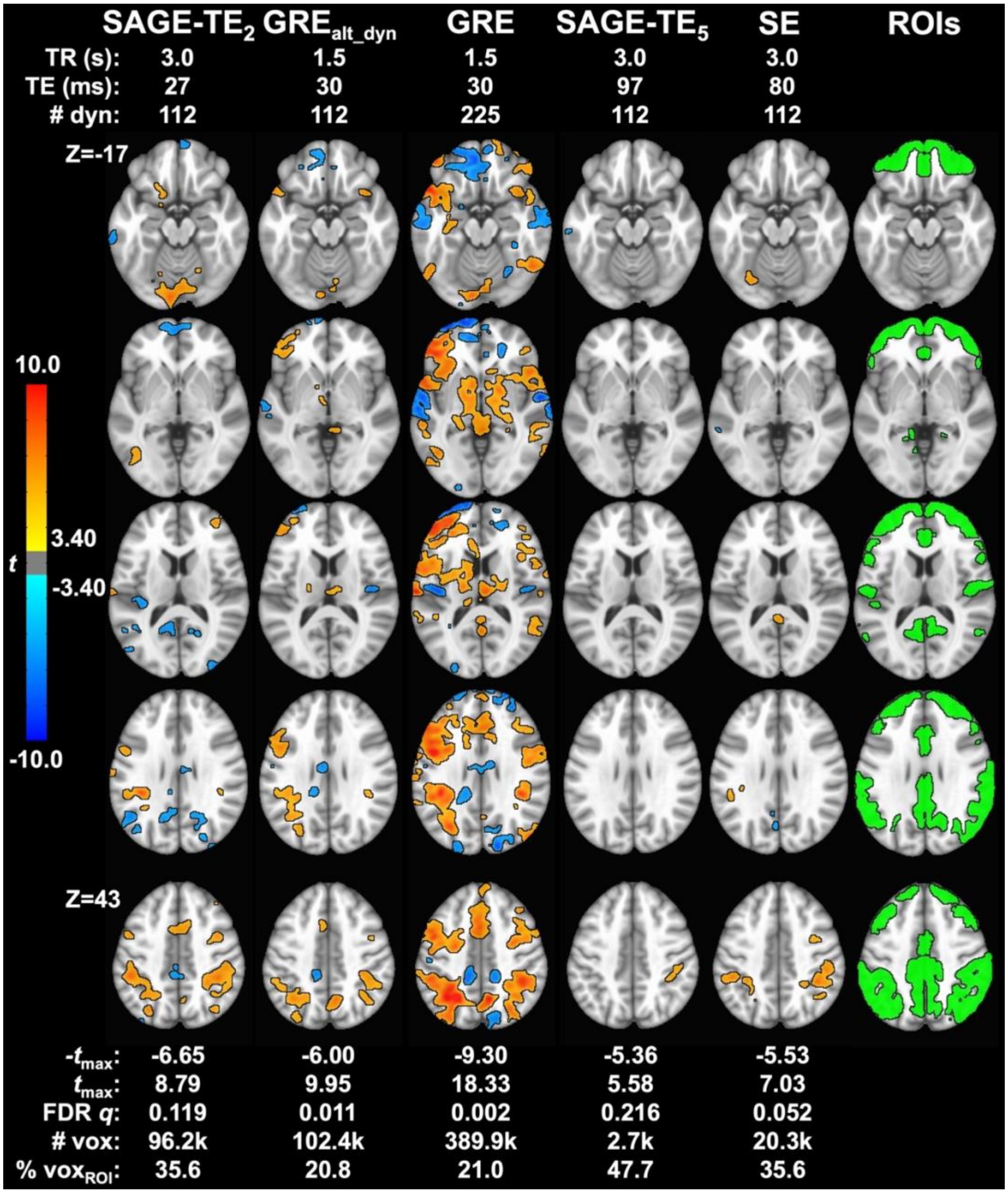

Figure S5. Group-level effect size maps for the working memory task across macro- (TE_2, SAGE T_2*, and SAGE wT_2^*) and microvascular (TE_5, SAGE T_2, and SAGE wT_2) acquisitions, thresholded for $|g| \geq 0.80$ (i.e., at least a large effect size). # vox = voxel count for significant functional activation.

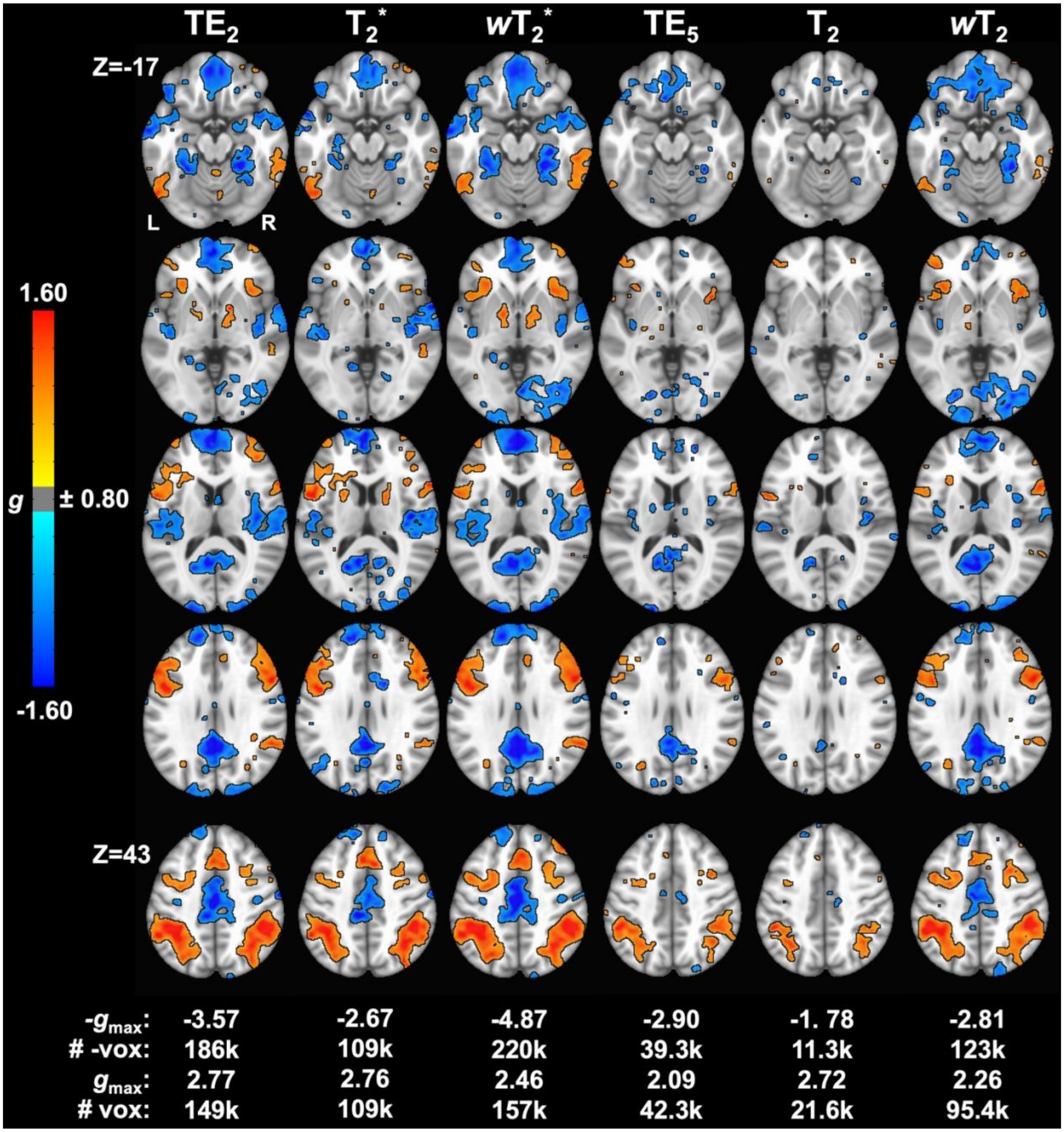

Figure S6. Group-level *t*-maps for the visual stimulus task across macro- (TE_2, SAGE T_2*, and SAGE wT_2^*) and microvascular (TE_5, SAGE T_2, and SAGE wT_2) acquisitions. # vox = voxel count for significant functional activation.

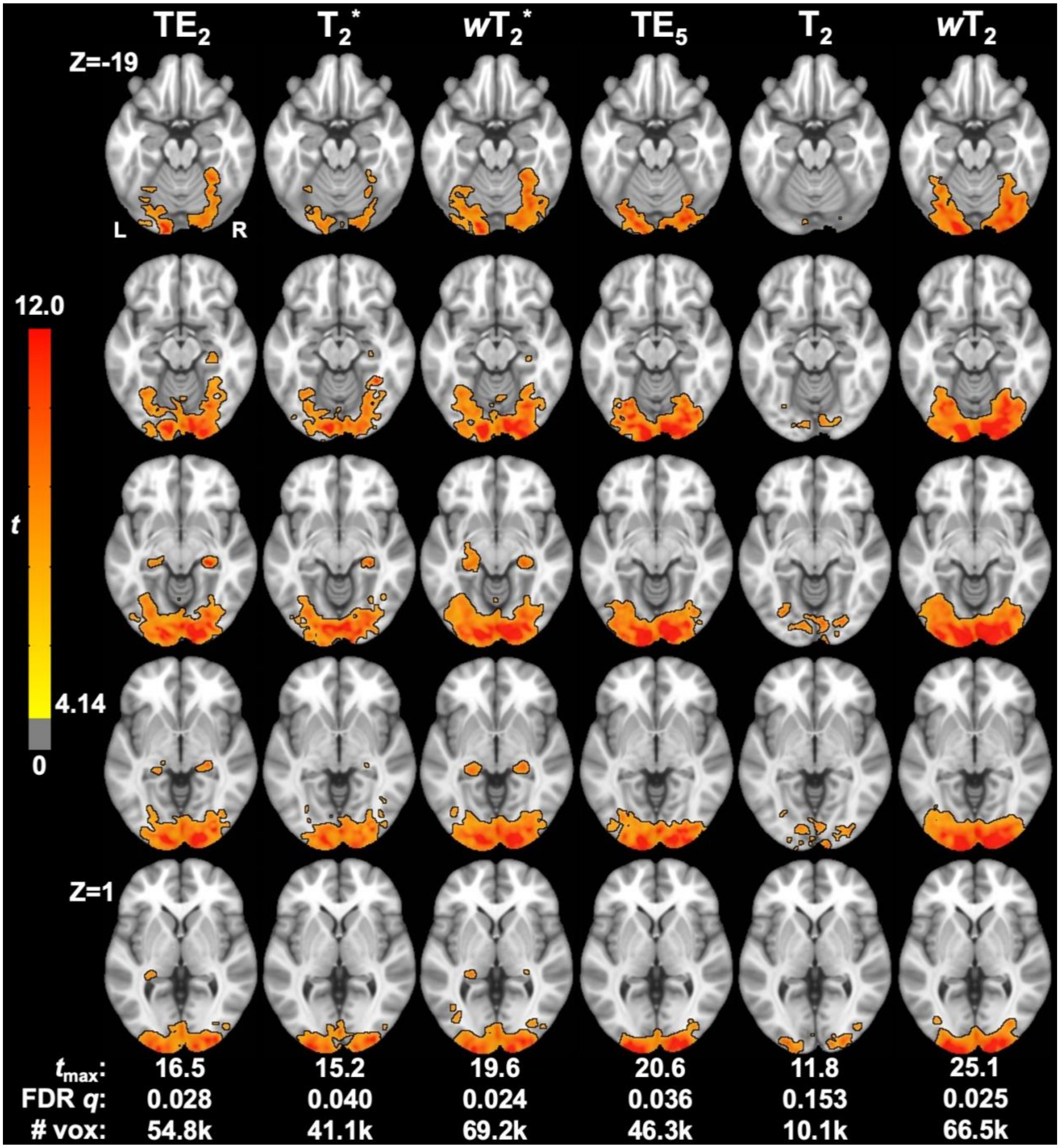

Figure S7. Group-level effect size maps for the visual stimulus task across macro- (TE_2, SAGE T_2*, and SAGE wT_2^*) and microvascular (TE_5, SAGE T_2, and SAGE wT_2) acquisitions, thresholded for $|g| \geq 0.80$ (i.e., at least a large effect size). # vox = voxel count for significant functional activation.

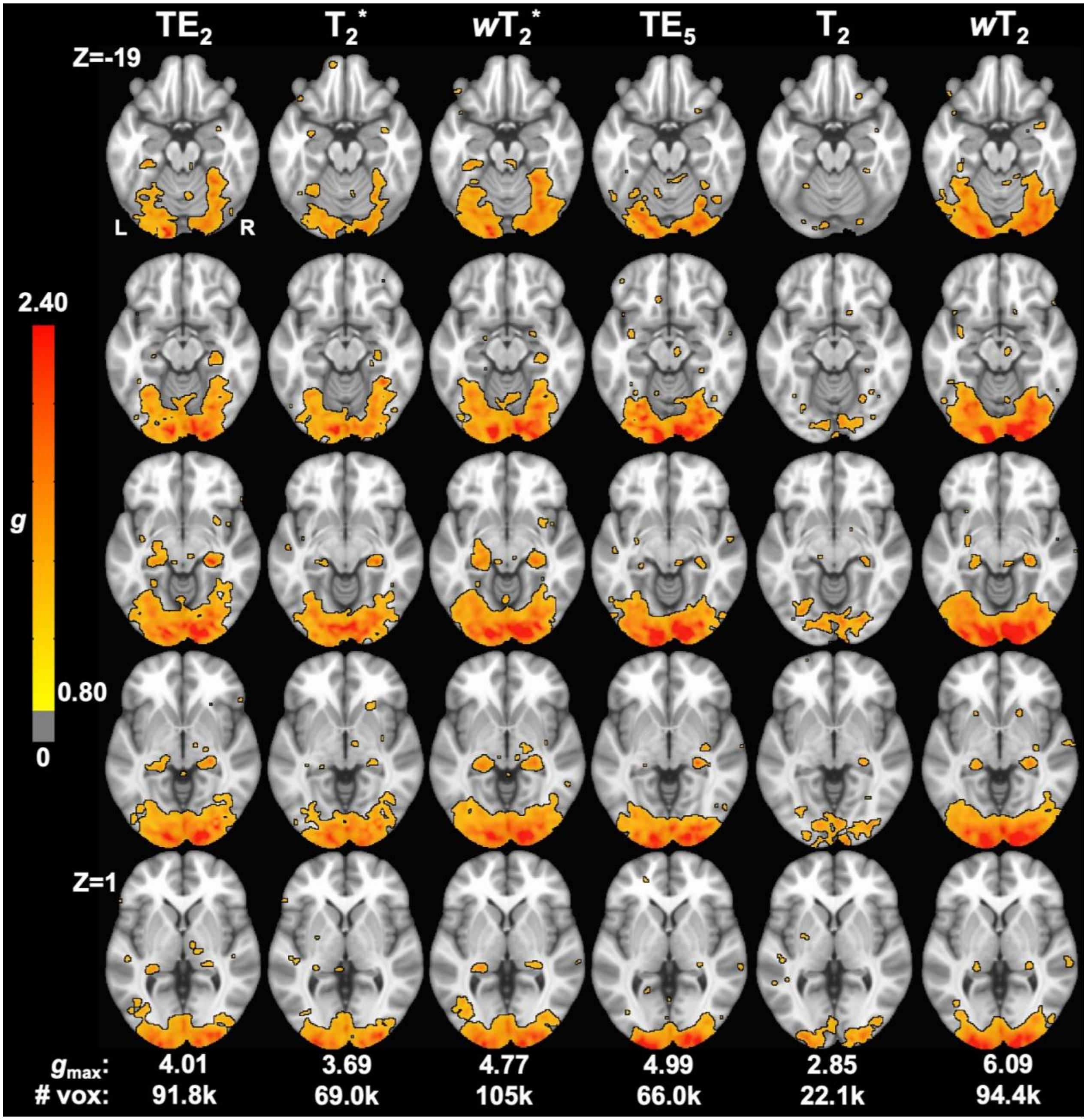

Table S1. Group-level mean and standard deviation (SD) T_2^* and T_2 values from the optimized SAGE-fMRI protocol, applied in the *N*-back working memory task, in whole brain and gray and white matter.

Parameter	Tissue	Mean ± SD
T_2^* (ms)	Whole Brain	47.26 ± 2.46
	Gray Matter	53.35 ± 3.34
	White Matter	51.29 ± 2.72
T_2 (ms)	Whole Brain	70.98 ± 3.27
	Gray Matter	77.51 ± 3.62
	White Matter	66.65 ± 4.04

Table S2. Group-level mean and standard deviation tSNR across optimization protocols for each echo time in gray and white matter.

TE	MB	SENSE	Gray Matter Mean ± SD	White Matter Mean ± SD
1	1	2	170. ± 22.3	232 ± 18.7
		2.5	162 ± 20.2	221 ± 20.4
		3	160. ± 15.4	205 ± 9.50
	2	2	103 ± 17.8	156 ± 21.3
		2.5	118 ± 14.4	161 ± 14.9
		3	104 ± 9.42	139 ± 12.5
	3	2	102 ± 12.7	127 ± 14.7
		2.5	85.2 ± 20.4	99.7 ± 17.4
		3	86.7 ± 10.8	92.2 ± 9.21
	4	2	100. ± 11.9	116.2 ± 14.0
		2.5	82.2 ± 12.3	82.5 ± 13.6
		3	67.5 ± 10.2	59.7 ± 9.11
2	1	2	98.0 ± 10.4	154 ± 9.17
		2.5	105 ± 12.5	159 ± 14.2
		3	108 ± 11.2	154 ± 7.44
	2	2	52.6 ± 14.6	90.5 ± 24.5
		2.5	67.1 ± 9.67	105 ± 13.4
		3	60.1 ± 7.21	93.3 ± 12.3
	3	2	59.7 ± 8.57	86.5 ± 12.0
		2.5	52.2 ± 13.6	70.6 ± 13.8
		3	52.4 ± 5.93	64.5 ± 9.21
	4	2	57.2 ± 8.50	73.3 ± 12.3
		2.5	48.7 ± 8.60	53.6 ± 10.7
		3	41.5 ± 7.42	39.3 ± 8.02
3	1	2	49.6 ± 9.12	75.3 ± 7.27
		2.5	54.1 ± 8.64	84.5 ± 8.22
		3	56.8 ± 9.87	84.0 ± 6.93

		2	24.6 ± 9.84	39.6 ± 14.4
	2	2.5	34.5 ± 6.61	49.6 ± 8.92
		3	31.2 ± 4.11	44.2 ± 6.39
		2	28.6 ± 4.90	37.4 ± 5.70
	3	2.5	25.8 ± 5.26	32.4 ± 6.14
		3	26.3 ± 3.95	29.6 ± 5.08
		2	23.6 ± 3.96	30.8 ± 5.55
	4	2.5	20.5 ± 3.72	22.7 ± 4.38
		3	17.2 ± 2.87	16.7 ± 3.54
4		2	46.6 ± 7.79	70.0 ± 5.79
	1	2.5	51.7 ± 7.85	76.1 ± 7.35
		3	56.5 ± 7.58	77.0 ± 5.67
		2	25.4 ± 9.75	34.8 ± 13.3
	2	2.5	35.6 ± 6.38	44.1 ± 8.09
		3	31.7 ± 3.57	38.7 ± 5.81
		2	30.1 ± 4.66	32.7 ± 5.45
	3	2.5	27.0 ± 5.55	28.5 ± 5.25
		3	27.2 ± 4.13	25.3 ±4.49
		2	24.9 ± 3.70	26.4 ± 5.22
	4	2.5	21.2 ± 3.43	18.8 ± 4.00
		3	18.1 ± 2.62	14.0 ± 2.92
5		2	40.8 ± 6.63	56.1 ± 4.59
	1	2.5	47.1 ± 6.73	65.8 ± 6.51
		3	50.3 ± 6.59	66.4 ± 5.33
		2	23.2 ± 8.12	27.6 ± 10.8
	2	2.5	32.2 ± 5.66	36.2 ± 6.45
		3	28.1 ± 3.01	31.7 ± 4.65
		2	27.1 ± 4.34	26.1 ± 4.43
	3	2.5	24.5 ± 4.95	23.4 ± 3.96
		3	24.3 ± 3.63	20.5 ± 3.49
	4	2	22.4 ± 3.45	20.7 ± 3.67
		2.5	19.0 ± 3.12	14.3 ± 3.15

	3	15.7 ± 2.28	10.7 ± 2.16

Table S3. Tukey's HSD post-hoc results for tSNR comparisons for each TE across acquisition protocols with feasible parameters (i.e., full brain coverage ≥120 mm, TE_1 < 10 ms, TE_5 < 100 ms, which includes the eight most accelerated protocols: MB2-S2.5 to MB4-S3); significant results are italicized. In gray matter, the MB2-S2.5 protocol had significantly higher tSNR than MB3-S2.5 and MB3-S3 for TE_1 only. In white matter, these differences were repeated across all TEs; additionally, MB2-S3 had significantly higher tSNR than MB3-S2.5 (TE_1) and MB3-S3 (all TEs). - not significant; * *p<0.05;* ** *p<0.01;* *** *p<0.001;* **** *p<0.0001*.

	**Acq. 1**	MB2 S2.5				MB2 S3			MB3 S2		MB3 S2.5
	**Acq. 2**	MB2 S3	MB3 S2	MB3 S2.5	MB3 S3	MB3 S2	MB3 S2.5	MB3 S3	MB3 S2.5	MB3 S3	MB3 S3
**GM**	**TE_1**	0.90<br>-	0.78<br>-	*0.021*<br>*	*0.034*<br>*	1.0<br>-	0.61<br>-	0.73<br>-	0.78<br>-	0.87<br>-	1.0<br>-
	**TE_2**	0.99<br>-	0.98<br>-	0.34<br>-	0.36<br>-	1.0<br>-	0.97<br>-	0.97<br>-	0.98<br>-	0.98<br>-	1.0<br>-
	**TE_3**	1.0<br>-	0.92<br>-	0.49<br>-	0.59<br>-	1.0<br>-	0.96<br>-	0.98<br>-	1.0<br>-	1.0<br>-	1.0<br>-
	**TE_4**	0.99<br>-	0.91<br>-	0.37<br>-	0.40<br>-	1.0<br>-	0.97<br>-	0.98<br>-	1.0<br>-	1.0<br>-	1.0<br>-
	**TE_5**	0.97<br>-	0.87<br>-	0.34<br>-	0.286<br>-	1.0<br>-	0.99<br>-	0.98<br>-	1.0<br>-	1.0<br>-	1.0<br>-
**WM**	**TE_1**	0.35<br>-	*0.011*<br>*	*1.5e-7*<br>****	*4.9e-9*<br>****	0.95<br>-	*1.7e-3*<br>**	*8.1e-5*<br>****	0.11<br>-	*0.011*<br>*	1.0<br>-
	**TE_2**	0.91<br>-	0.36<br>-	*1.2e-3*<br>**	*6.3e-5*<br>****	1.0<br>-	0.13<br>-	*0.014*<br>*	0.61<br>-	0.15<br>-	1.0<br>-
	**TE_3**	0.98	0.19	*8.0e-3*	*9.2e-4*	0.91	0.22	*0.047*	0.99	0.79	1.0

		-	-	**	***	-	-	*	-	-	-
	**TE_4**	0.96<br>-	0.14<br>-	*7.1e-3*<br>**	*4.2e-4*<br>***	0.91<br>-	0.27<br>-	*0.037*<br>*	0.99<br>-	0.74<br>-	1.0<br>-
	**TE_5**	0.95<br>-	0.07<br>-	*5.7e-3*<br>**	*2.6e-4*<br>***	0.81<br>-	0.25<br>-	*0.029*<br>*	0.99<br>-	0.81<br>-	1.0<br>-

Table S4. *N*-back working memory task tSNR, CNR, and *t*-score ROI analyses across macro- (TE2, SAGE T_2*, and SAGE wT_2^*) and microvascular (TE5, SAGE T_2, and SAGE wT_2) acquisitions. A visualization of the ROIs can be found in Figure 6. For CNR, "Max" indicates the maximum value (whether positive for activation or negative for deactivation, indicated by sign). IFG = Inferior Frontal Gyrus; FP = Frontal Pole; MFG = Middle Frontal Gyrus; PL = Parietal Lobe; CGa = Cingulate Gyrus anterior division; CGp = Cingulate Gyrus posterior division; FMC = Frontal Medial Cortex.

Statistic	ROI	Image	Mean	SD	Max
tSNR	IFG (6.2k vox)	TE_2	229.5	18.0	285.9
		T_2^*	90.7	8.6	119.2
		wT_2^*	277.7	23.3	372.0
		TE_5	138.2	12.4	174.8
		T_2	97.3	14.1	126.6
		wT_2	185.1	15.3	222.9
	FP (80.0k vox)	TE_2	190.9	41.1	306.4
		T_2^*	78.2	13.3	126.4
		wT_2^*	250.1	46.2	409.5
		TE_5	105.4	12.2	145.9
		T_2	60.7	19.9	114.4
		wT_2	141.7	19.8	196.2
	MFG (16.0k vox)	TE_2	203.5	30.4	291.3
		T_2^*	73.0	15.4	112.2
		wT_2^*	256.4	38.0	394.4
		TE_5	127.0	9.0	153.8
		T_2	60.2	22.0	112.2
		wT_2	162.7	13.7	200.7
	PL (180.3k vox)	TE_2	243.4	46.5	387.4
		T_2^*	90.1	21.5	154.8
		wT_2^*	300.5	60.9	545.1
		TE_5	143.6	17.5	193.6
		T_2	92.2	30.9	153.4

		wT_2	189.7	23.8	277.8
	CGa (12.6k vox)	TE_2	206.8	31.6	297.3
		T_2^*	76.4	9.8	116.0
		wT_2^*	251.5	39.8	364.4
		TE_5	114.8	9.8	123.2
		T_2	69.2	12.5	96.3
		wT_2	158.6	14.5	189.4
	CGp (10.8k vox)	TE_2	219.4	39.4	294.6
		T_2^*	81.2	10.9	105.5
		wT_2^*	274.1	41.7	380.4
		TE_5	118.2	12.8	139.4
		T_2	84.6	14.5	109.3
		wT_2	164.9	17.6	196.8
	FMC (4.7k vox)	TE_2	117.7	33.2	203.2
		T_2^*	71.8	12.2	112.5
		wT_2^*	192.2	29.1	293.9
		TE_5	101.2	119.6	12.3
		T_2	35.6	13.2	73.0
		wT_2	128.4	21.4	158.8
CNR	IFG (6.2k vox)	TE_2	0.4	0.2	1.0
		T_2^*	0.4	0.2	0.9
		wT_2^*	0.5	0.3	1.1
		TE_5	0.2	0.1	0.5
		T_2	0.1	0.1	0.5
		wT_2	0.4	0.2	0.8
	FP (80.0k vox)	TE_2	0.3	0.2	0.9
		T_2^*	0.3	0.2	0.8
		wT_2^*	0.3	0.2	0.9
		TE_5	0.1	0.1	0.5
		T_2	0.1	0.1	0.4
		wT_2	0.2	0.1	0.7

	MFG (16.0k vox)	TE_2	0.5	0.3	1.2
		T_2^*	0.4	0.2	1.1
		wT_2^*	0.5	0.3	1.2
		TE_5	0.2	0.1	0.6
		T_2	0.2	0.1	0.6
		wT_2	0.3	0.2	0.8
	PL (180.3k vox)	TE_2	0.5	0.4	1.5
		T_2^*	0.5	0.3	1.4
		wT_2^*	0.6	0.4	1.6
		TE_5	0.2	0.2	0.8
		T_2	0.2	0.1	0.8
		wT_2	0.4	0.2	1.2
	CGa (12.6k vox)	TE_2	-0.3	0.2	-0.8
		T_2^*	-0.2	0.1	-0.6
		wT_2^*	-0.3	0.2	-0.9
		TE_5	-0.1	0.1	-0.4
		T_2	-0.1	0.1	-0.3
		wT_2	-0.2	0.1	-0.6
	CGp (10.8k vox)	TE_2	-0.5	0.2	-0.9
		T_2^*	-0.4	0.1	-0.8
		wT_2^*	-0.5	0.2	-1.0
		TE_5	-0.2	0.1	-0.6
		T_2	-0.1	0.1	-0.4
		wT_2	-0.4	0.2	-0.9
	FMC (4.7k vox)	TE_2	-0.7	0.2	-1.0
		T_2^*	-0.6	0.2	-0.8
		wT_2^*	-0.7	0.1	-1.1
		TE_5	-0.3	0.1	-0.6
		T_2	-0.2	0.1	-0.5
		wT_2	-0.5	0.1	-0.7
t	IFG	TE_2	5.1	0.7	8.2

(6.2k vox)	T_2^*	5.2	0.8	8.8
	wT_2^*	5.2	0.8	7.8
	TE_5	-	-	-
	T_2	-	-	-
	wT_2	4.9	0.6	6.9
FP (80.0k vox)	TE_2	-5.2 | 5.5	0.8 | 1.1	-8.2 | 10.9
	T_2^*	-5.2 | 5.5	0.9 | 1.0	-9.3 | 9.4
	wT_2^*	-5.3 | 5.4	0.9 | 0.9	-9.4 | 8.6
	TE_5	- | -	- | -	- | -
	T_2	- | -	- | -	- | -
	wT_2	-5.1 | -	0.9 | -	-9.3 | -
MFG (16.0k vox)	TE_2	4.9	0.7	7.7
	T_2^*	5.0	0.9	8.6
	wT_2^*	4.9	0.7	8.0
	TE_5	4.8	0.6	7.1
	T_2	-	-	-
	wT_2	5.5	0.8	7.6
PL (180.3k vox)	TE_2	5.3	1.0	9.5
	T_2^*	5.2	0.8	9.6
	wT_2^*	5.4	1.0	10.1
	TE_5	4.9	0.6	8.1
	T_2	5.0	0.9	11.2
	wT_2	5.1	0.8	9.0
CGa (12.6k vox)	TE_2	-5.5	1.2	-11.9
	T_2^*	-5.1	0.8	-9.1
	wT_2^*	-5.9	1.5	-12.0
	TE_5	-	-	-
	T_2	-	-	-
	wT_2	-5.0	0.7	-7.7
CGp	TE_2	-6.1	1.5	-14.2
	T_2^*	-5.8	1.3	-11.0

	(10.8k vox)	wT_2^*	-6.0	1.4	-13.0
		TE_5	-5.3	1.1	-11.9
		T_2	-4.7	0.6	-6.8
		wT_2	-6.0	1.3	-11.6
	FMC (4.7k vox)	TE_2	-4.9	0.6	-8.0
		T_2^*	-4.7	0.5	-7.5
		wT_2^*	-5.0	0.6	-7.7
		TE_5	-5.0	0.8	-8.0
		T_2	-	-	-
		wT_2	-4.7	0.4	-6.4
